# HER4 Affects Sensitivity to Tamoxifen and Abemaciclib in Luminal Breast Cancer Cells and Restricts Tumor Growth in MCF-7-Based Humanized Tumor Mice

**DOI:** 10.3390/ijms25137475

**Published:** 2024-07-08

**Authors:** Veruschka Albert, Christina Bruss, Deniz Tümen, Gerhard Piendl, Florian Weber, Edgar Dahl, Stephan Seitz, Olaf Ortmann, Anja K. Wege, Gero Brockhoff

**Affiliations:** 1 Department of Gynecology and Obstetrics, University Medical Center Regensburg, 93935 Regensburg, Germany; veruschka.albert@ukr.de (V.A.); christina.bruss@ukr.de (C.B.); gerhard.piendl@ukr.de (G.P.); sseitz@csj.de (S.S.); oortmann@csj.de (O.O.); anja.wege@ukr.de (A.K.W.); 2 Bavarian Cancer Research Center (BZKF), 93053 Regensburg, Germany; deniz.tuemen@ukr.de (D.T.); florian.weber@ukr.de (F.W.); 3 Department of Internal Medicine I, Gastroenterology, Hepatology, Endocrinology, Rheumatology and Infectious Diseases, University Hospital Regensburg, 93053 Regensburg, Germany; 4 Institute of Pathology, University of Regensburg, 93093 Regensburg, Germany; 5 Institute of Pathology, Medical Faculty, RWTH Aachen University, 52074 Aachen, Germany; edahl@ukaachen.de

**Keywords:** hormone receptor positive (HR^+^) breast cancer, human epidermal growth factor receptor related (HER4), tamoxifen, abemaciclib, humanized tumor mice (HTM)

## Abstract

The impact of the HER4 receptor on the growth and treatment of estrogen receptor-positive breast cancer is widely uncertain. Using CRISPR/Cas9 technology, we generated stable HER4 knockout variants derived from the HER4-positive MCF-7, T-47D, and ZR-75-1 breast cancer cell lines. We investigated tumor cell proliferation as well as the cellular and molecular mechanisms of tamoxifen, abemaciclib, AMG232, and NRG1 treatments as a function of HER4 in vitro. HER4 differentially affects the cellular response to tamoxifen and abemaciclib treatment. Most conspicuous is the increased sensitivity of MCF-7 in vitro upon HER4 knockout and the inhibition of cell proliferation by NRG1. Additionally, we assessed tumor growth and immunological effects as responses to tamoxifen and abemaciclib therapy in humanized tumor mice (HTM) based on MCF-7 HER4-wildtype and the corresponding HER4-knockout cells. Without any treatment, the enhanced MCF-7 tumor growth in HTM upon HER4 knockout suggests a tumor-suppressive effect of HER4 under preclinical but human-like conditions. This phenomenon is associated with an increased HER2 expression in MCF-7 in vivo. Independent of HER4, abemaciclib and tamoxifen treatment considerably inhibited tumor growth in these mice. However, abemaciclib-treated hormone receptor-positive breast cancer patients with tumor-associated mdm2 gene copy gains or pronounced HER4 expression showed a reduced event-free survival. Evidently, the presence of HER4 affects the efficacy of tamoxifen and abemaciclib treatment in different estrogen receptor-positive breast cancer cells, even to different extents, and is associated with unfavorable outcomes in abemaciclib-treated patients.

## 1. Introduction

Both oncogenic and tumor-suppressive activity have been attributed to the human epidermal growth factor receptor related (HER4) receptor when expressed in breast cancer (BC) cells [1]. While a prognostically favorable impact of HER4 has been primarily observed in HER2-positive and triple-negative BC, an unfavorable effect has been associated with estrogen receptor (ESR)-positive (i.e., luminal) BC [2]. Subtype-specific effects are triggered by ligand-dependent and -independent receptor activation that triggers many-sided intracellular signaling [3,4]. Growth factor binding elicits, primarily, receptor phosphorylation and interaction between related receptors (HER1, 2, and 3), whereas a twostep shedding process of HER4 by disintegrin and metalloprotease 17 (ADAM17) and γ-secretase results in the release of an extra- and intracellular domain (4ECD and 4ICD), respectively [5,6,7]. (Of note, only the cleavable and not the non-cleavable HER4 receptor isoforms are expressed in malignant epithelial breast cells [2]). 4ICD has been found to have a pro-apoptotic, pro-differentiation, or pro-proliferative effect [2,8,9,10,11,12]. Accordingly, in non-malignant tissues, HER4/4ICD may act as a regulator in these processes, and as a result of the disruption of this balance, HER4-regulated processes might contribute to tumorigenesis.

Viewed across subtypes, HER4 has been repeatedly shown to be increasingly expressed in ESR-positive BC compared to other BC entities [2], and it can be activated under the influence of the steroid hormone estrogen [6]. It has been repeatedly reported that a pro-proliferative and, thus, tumor-promoting effect predominates in luminal BC cells and this is, with reasonable certainty, due to a variety of molecular mechanisms that directly and indirectly stimulate the transcriptional activity of the ESR. Thus, HER4 has an exceptional functional importance in luminal BC [1,10,13,14,15]. Nevertheless, reports on the prognostic impact of HER4 in luminal BC are partially inconsistent and a tumor-suppressive impact of HER4 in (luminal) BC has also been reported both in the experimental [16] and the clinical setting [17].

Due to the capacity of HER4 to interfere with a variety of intracellular signaling cascades, it is not surprising that the receptor tyrosine kinase also manipulates molecular treatment effects and modulates therapy efficiencies. For example, we retrospectively associated higher HER4 expression with a decreased sensitivity to tamoxifen treatment and a reduced overall survival of postmenopausal women who suffered from ESR-positive BC [18]. Considering a number of treatment-related studies, HER4 seems to favorably or unfavorably predict a variety of BC therapies [19,20], including endocrine treatments (with, e.g., the anti-estrogen tamoxifen or related substances). This kind of therapy represents the mainstay for the management of ESR-positive BC; however, rather novel treatment regimens have been implemented in clinics as well. CDK4/6 targeting has evolved to become another stable pillar for the maintenance of disease remission in the adjuvant setting. CDK4/6 inhibitors (CDK4/6i), namely, ribociclib and palbociclib, but, in particular, abemaciclib, turned out to be particularly effective in luminal BC but not in other BC subtypes [21,22]. This is due to the estrogen/ESR system that drives the CDK4/6 and Cyclin-D1 expression and interaction [23]. However, similar to endocrine therapies, resistance to CDK4/6 inhibition poses a frequent and, thus, serious clinical issue that can be attributed to intrinsic or acquired mechanisms [24].

Other novel treatments for luminal BC are still under evaluation, amongst them is the specific targeting of the murine double minute 2 (mdm2) protein, otherwise designated as human double minute 2 (hdm2 [25]). As an E3 ubiquitin ligase, mdm2 represents the key regulator of p53 and, thus, controls the balance between cell proliferation and apoptotic cell death [26]. Mechanistically, protein overexpression of mdm2 (which is typically due to a corresponding gain of gene copies) and enhanced mdm2 activity causes a pronounced p53 ubiquitination that results in the degradation of this prominent and, in BC, relevant tumor suppressor. p53 deprivation entails both the loss of cell cycle control and the reduced capacity of cells to undergo programmed cell death. The molecular mechanisms exerted by mdm2/p53 are well known [26,27,28,29,30] and mdm2 has been described to be involved in the genesis and progression of BC in general [31,32,33]. Relatively new, however, is our finding of *mdm2* gene amplification in about 10% of luminal BCs, which is associated with a poor course and outcome of the disease [34]. A variety of mdm2 inhibitors are being tested in the preclinical and early clinical setting [35]. However, this strategy could not yet be translated into clinical practice.

The exploitation of the HER4 expression as prognostic marker, as predictor for target-specific or other (systemic) treatments, or as an independent therapeutic target is still in a quite immature state. Nevertheless, a considerable amount of data suggests the trilateral communication and cross-signaling of HER4, CDK4/6, and mdm2—in particular, in ESR-positive BC. In particular, mdm2 is considered to serve as a highly relevant predictive marker for an anit-CDK4/6 therapy [36,37]. However, the interdependence of these molecules has not yet been clarified in detail.

Here, we analyzed relevant but insufficiently explored treatment modalities for ESR-positive BC considering HER4, CDK4/6, and mdm2. More specifically, we used MCF-7, T-47D, and ZR-75-1 BC cells as simple but well-characterized ESR- and HER4-positive BC models [38,39]. We evaluated treatment efficiencies and the molecular mechanisms involved in CDK4/6- and mdm2-targeting using abemaciclib and AMG232, respectively. In addition, the cells were treated with tamoxifen in both the absence and presence of the HER3/HER4 receptor-specific ligand neugregulin-1 (NRG1). Moreover, certain specified combination treatments (abemaciclib plus AMG232 and tamoxifen plus NRG1) were tested. In order to assess the impact of HER4 on aforementioned treatments, we generated stable HER4 knockout clones from the respective cell lines using CRISPR/Cas9 technology. Extending the translational value of these analyses, we generated humanized tumor mice (HTM) [40] based on MCF-7 wild type (WT) and MCF7 knock out (KO) cells for tamoxifen and abemaciclib treatment studies as a function of HER4. This mouse model not only facilitates the analysis of tumor growth under human-like conditions but also allows evaluating a potential human immune response involved in individual treatments.

## 2. Results

### 2.1. Cell Cycle Kinetics in Luminal HER4 WT and Successfully Generated HER4 KO Cells as a Function of Treatment

HER4 KO cell lines derived from MCF-7, T-47D, and ZR-75-1 cells were generated by CRISPR-Cas9 technology. The ribonucleoprotein complex (consisting of the sgRNA and the Cas9 protein) was built and transfected into the cells by electroporation and cell clones were expanded and screened (Supplementary Figure S1A). Loss of HER4 expression was determined by flow cytometry (Supplementary Figure S1B) and Western blotting (Supplementary Figure S1C). Upon sequencing (Supplementary Figure S1D), one particular clone for each cell line was identified and expanded. HER4 gene knockout (KO) was highly specific in all cell lines, as none of the structurally related HER receptors were markedly impaired in expression (Supplementary Figure S1E). The ESR expression was downregulated in MCF-7 cells and nearly completely switched off in ZR-75-1 cells as a consequence of HER4 knockout. However, the proliferative capacity did not differ between WT and KO cells (Supplementary Figure S1F).

**Figure 1:**
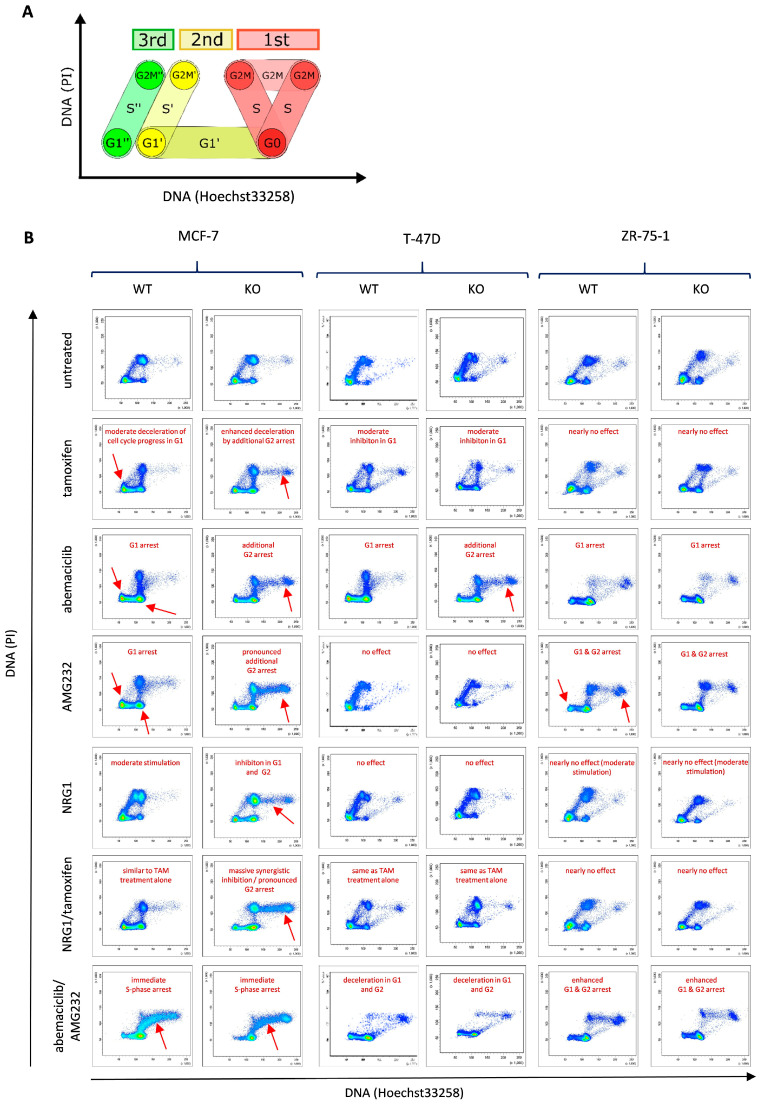
Dynamic proliferation assessment by flow cytometry. (**A**) The diagram illustrates the course of a non-synchronized cell population and sub-cohorts within three successive cell cycles (red 1st, color 2nd, and green 3rd) monitored within a period of 96 h. (**B**) Real flow cytometry measurements recorded at 96 h upon continuous treatments. MCF-7, T-47D, and ZR-75-1 WT or KO cells were alternatively treated with tamoxifen, abemaciclib, AMG232, NRG1, NRG1/tamoxifen, or abemaciclib/AMG232. Annotations in red indicate the most pronounced treatment-induced effects. Red arrows point at the most pronounced effects. The complete panels of measurements done after 8, 16, 24, 48, and 72 h, respectively, are shown in Supplementary Figure S2.

Cell cycle kinetics were quantitatively monitored over a period of 96 h by flow cytometry. Figure 1A illustrates the pattern derived from cells attributed to three successive cell cycles, including cell cycle phases G1, S, and G2/M. In Figure 1B, a compilation of real flow-cytometric snapshot measurements taken after 96 h is displayed. The inserted annotations mark the most prominent effects. In order to display the total course of cell cycle progress, all measurements over the period of 96 h in appropriate time intervals are included in the Supplementary Material (Supplementary Figure S2A–C). The efficiencies of the different treatments on the three cell lines at the different time points are also visualized as so-called “exit curves”. Cell cohorts, which have not yet left the first cell cycle under given conditions, are quantitatively displayed in Supplementary Figure S3.

The HER4 knockout sensitizes MCF-7 cells to all treatments, as evidenced by a pronounced G1- and an additional G2-phase arrest. Interestingly, the combined abemaciclib/AMG232 treatment caused an abrupt halt of the cell cycle progress in the S-phase of the first cell cycle at the moment of substrate addition. Strikingly, the addition of NRG1 to MCF-7 WT cells stimulated the cell cycle progress and, thus, the proliferation speed, whereas MCF-7 KO cells were inhibited when exposed to NRG1.

T-47D cells were moderately inhibited by the exposition to tamoxifen, whereas the treatment with abemaciclib was much more efficient. These cells did not show any response to the treatment with AMG232 or NRG1, respectively. Like with the MCF-7 cells, the sensitivity to abemaciclib treatment was enhanced upon HER4 knockout, which is due to an additional G2 arrest. The exposure to NRG1, either alone or in combination with tamoxifen, had no effect on T-47D cell proliferation, independently from the presence or absence of HER4.

Interestingly, ZR-75-1 cells were basically refractory to the tamoxifen treatment both with and without HER4 expression. However, this cell line was highly sensitive when treated with abemaciclib and AMG232, applied separately or in combination. The cell proliferation of ZR-75-1 WT or KO cells was not measurably affected in the presence of NRG1.

Overall, treatment effects vary in the three ER-positive BC cell lines, while MCF-7 cells appeared most sensitive and treatment effects were even enhanced by the HER4 knockout. The addition of the growth factor NRG1 is an exception: A growth stimulating effect in MCF-7 cells was reversed upon the HER4 knockout.

### 2.2. HER4 Knockout Sensitizes MCF-7 Cells to Long-Term Tamoxifen and Abemaciclib Treatment

**Figure 2:**
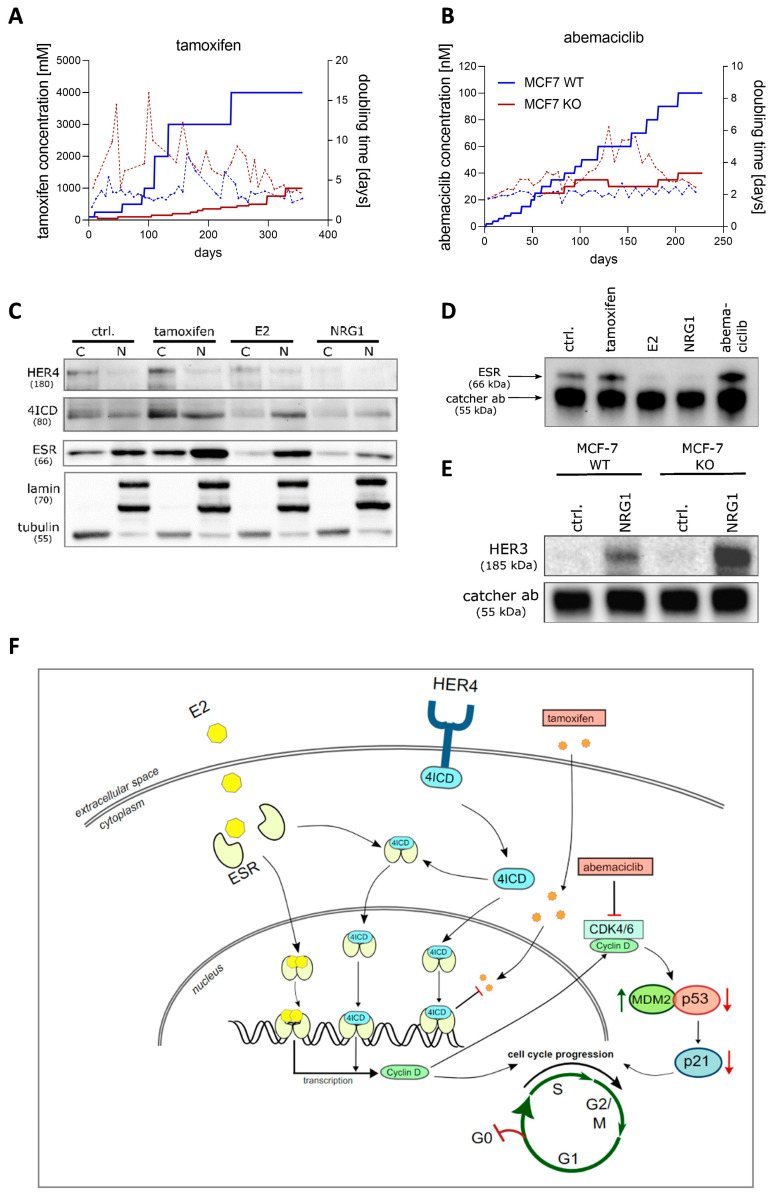
Effects of HER4 knock-out on MCF-7 cells in vitro. (**A**) Long-term treatment of MCF-7 WT (soild blue line) and MCF-7 KO (soild red line) cells with tamoxifen and corresponding cell doubling times (dashed lines) as a function of concentration. (**B**) Abemaciclib long-term treatment of MCF-7 WT and KO cells and corresponding cell doubling times (dashed lines) as a function of concentration. (**C**) Analysis of HER4, 4ICD, and ESR cytoplasmic and nuclear localization by Western blot using fractionated protein lysates (C = cytosol; N = nucleus) of MCF-7 WT cells untreated (ctrl.) and after treatment with tamoxifen (1 µM), E2 (100 nM), or NRG1 (30 ng/mL) for 24 h, respectively. Lamin (nucleus) and tubulin (cytosol) were used as loading controls and indicate the purity of the respective fractions. (**D**) Immunoprecipitation analysis by Western blotting of untreated (ctrl.) and 24 h tamoxifen (1 µM), E2 (100 nM), NRG1 (30 ng/mL), and abemaciclib (100 nM) treated MCF-7 WT cells. Immunoprecipitation was performed using a rabbit anti-HER4 catcher antibody and interaction of HER4/4ICD was analyzed by using a rabbit anti-ESR detection antibody. (**E**) Immunoprecipitation by Western blotting of untreated (ctrl.) and NRG1 (30 ng/mL)-treated MCF-7 WT and KO cells for 6 h. HER3 was detected by using a mouse anti-HER3 antibody upon HER2 immunoprecipitation. (**F**) A supposed model of HER4/4ICD interplay in the presence of E2, tamoxifen, or abemaciclib. Black arrows indicate molecule cross-linking. Red arrows indicate molecule downregulation.

To evaluate the effect of HER4 on long-term sensitivity, MCF-7 WT and KO cells were constantly exposed to increasing concentrations of tamoxifen and abemaciclib, respectively. Compared to MCF-7 WT cells, we found a higher sensitivity to tamoxifen in MCF-7 KO cells upon HER4 knockout, as these cells showed increased cell doubling times when exposed to low tamoxifen concentrations from the outset (Figure 2A). The persistently pronounced sensitivity of MCF-7 KO cells to tamoxifen treatment is not due to an acquired ESR mutation. Codon-specific sequencing revealed no mutations within the most relevant exon 8 codons (i.e., # 524, 536, 537, 538) in WT nor in KO cells after 1 year of treatment. Similar effects were seen when the cells were exposed to abemaciclib; however, higher sensitivity only became apparent after a treatment period of approximately 50 days. MCF-7 cells adapted quickly to rising abemaciclib concentrations, whereas, upon HER4 knockout, the cell doubling time was markedly increased (Figure 2B).

### 2.3. HER4/4ICD Receptor Localization and Interaction with ERS in Function of Treatment

Western blot data of fractionated protein lysates revealed both a HER4/4ICD and an ESR upregulation in response to tamoxifen. On the contrary, NRG1 exposure led to a downregulation of both HER4/4ICD and ESR (Figure 2C). Upon exposure to E2, an increased 4ICD expression was observed in the cell nucleus, indicating a HER4 receptor cleavage and, subsequently, 4ICD nuclear translocation as the expression of the entire HER4 receptor was decreased (Figure 2C). In the presence of E2, the ESR was displaced into the cell nucleus (Figure 2C). Interestingly, the immunoprecipitation data, which evidenced an interaction of HER4/4ICD and ESR under untreated conditions, showed a decreased interplay after E2 and NRG1 treatment. In contrast, tamoxifen and abemaciclib increased HER4/4ICD—ESR interaction (Figure 2D). After NRG1 exposure, HER2 and HER3 receptor interaction was induced in both MCF-7 WT and KO cells but was found to be more pronounced in MCF-7 KO cells (Figure 2E). Figure 2F illustrates the supposed interplay between HER4/4ICD and the ESR in the presence of E2, tamoxifen, or abemaciclib.

### 2.4. The Absence of HER4 Increases Tumor Growth In Vivo and Modulates Treatment Efficiency of Abemaciclib and Tamoxifen in HTM

**Figure 3:**
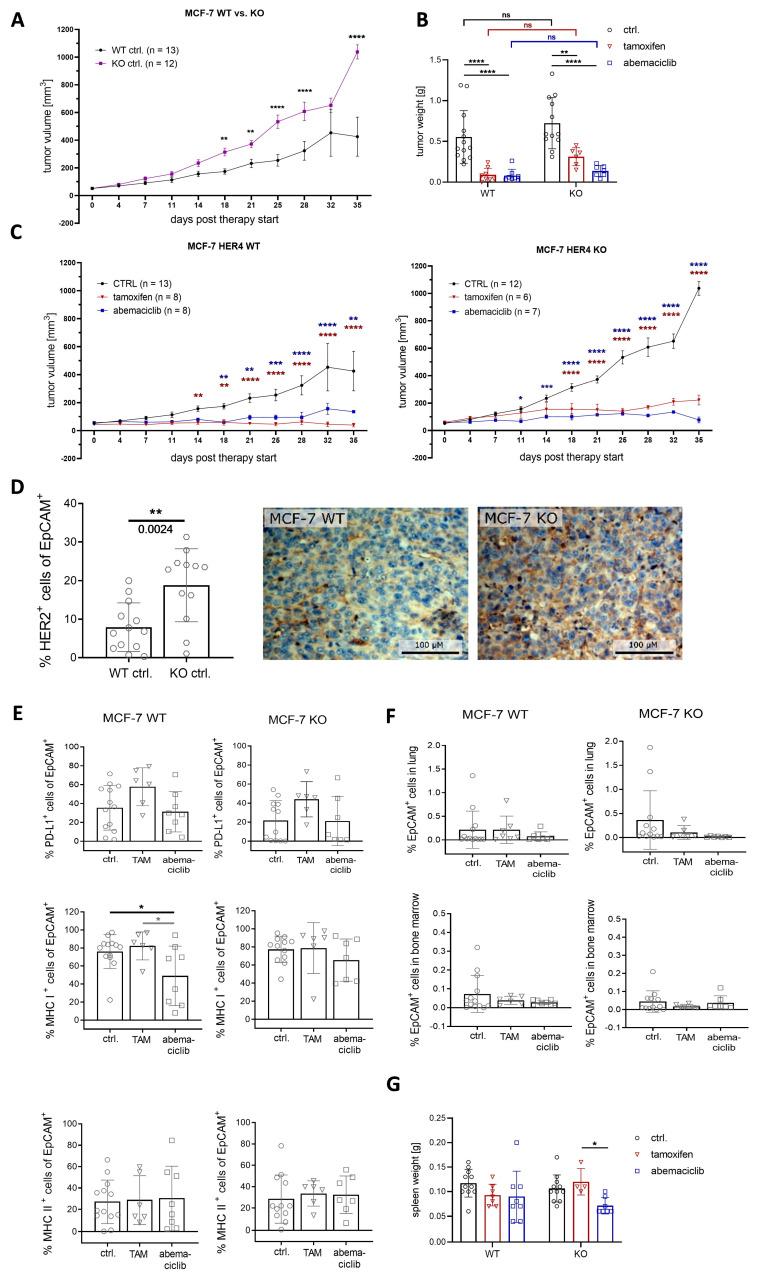
Tumor growth and tumor cell characterization in MCF-7-based HER4 WT and KO HTM. (**A**) Tumor growth of untreated HTM transplanted with MCF-7 WT or KO cells is shown. Data are presented as mean ± SD and two-way ANOVA and Sidak’s multiple comparisons tests were applied. (**B**) Tumor weight at the end of therapy is shown. Two-way ANOVA, Sidak’s, and Dunnett’s multiple comparisons tests were performed. (**C**) Tumor growth of HTM transplanted with MCF-7 WT or KO cells, either untreated (ctrl.) or treated with tamoxifen or abemaciclib, is displayed. Data were analyzed by two-way ANOVA and Dunnett’s multiple comparisons test. (**D**) Single tumor cells were analyzed by flow cytometry and the percentage of HER2 positive cells (of EpCAM pos. cells) is shown. Statistics were performed by unpaired *t* test, *p* = 0.0024. Immunohistochemical staining for HER2 was performed and is exemplarily shown for a MCF-7 WT and KO tumor, respectively. (**E**) PD-L1, MHC-I, and MHC-II expression on EPCAM^+^ cells is presented and data were analyzed by one-way ANOVA and Tukey’s multiple comparisons test. (**F**) Tumor cell dissemination in the lung and bone marrow (BM) was assessed by flow cytometry staining of EpCAM positive cells. (**G**) Spleen weight at the end of therapy is shown and differences were analyzed by two-way ANOVA, Sidak’s, and Dunnett’s multiple comparisons tests. (**B**,**D**–**G**) Data are shown as mean ± SD and number of animals are indicated by individual symbols. Statistical significances: * *p* ≤ 0.05, ** *p* ≤ 0.01 and *** *p* ≤ 0.001, **** *p* ≤ 0.0001.

In order to investigate the impact of the HER4 receptor on tumor growth and in the context of anti-hormonal and CDK4/6i therapy, humanized mice were transplanted with MCF-7 WT and MCF-7 HER4 knockout cells and treated with tamoxifen and abemaciclib, respectively (Supplementary Figure S4A). The absence of the HER4 receptor in untreated HER4 knockout mice led to accelerated tumor growth and, subsequently, to overall larger tumors compared to HER4 WT mice from day 18 (Figure 3A). These findings are consistent with the tumor weight at the end of therapy, which was 23.3% greater in untreated KO mice (Figure 3B). Treatment with tamoxifen reduced tumor growth in both groups. However, tamoxifen treatment completely inhibited tumor growth in WT mice compared to KO mice, which showed a slight increase in tumor volume (Figure 3C) and, thus, a 71.5% higher tumor weight at the end of treatment (Figure 3B). Treatment with abemaciclib in HER4 KO mice led to significantly reduced tumor growth from day 11 of therapy and efficiently blocked tumor growth during the treatment period (Figure 3C). Treatment with abemaciclib seemed to be even more effective than tamoxifen in these mice in terms of tumor volume, but also in tumor weight, which decreased down to 19.0% compared to controls (Figure 3B).

Interestingly, the phenotyping of tumor cells revealed an increased presence of HER2-positive cells in the absence of HER4 (Figure 3D). However, no significant changes were observed after treatment compared to control mice. Tamoxifen slightly induced PD-L1 expression on tumor cells independent of HER4 receptor status, whereas PD-L1 expression was not altered when mice were treated with abemaciclib (Figure 3E). MHC I expression was reduced by abemaciclib treatment in both WT and KO tumor mice, and was significant in MCF-7 WT mice. However, some mice exhibited barely any changes compared to the control group while others showed a strong decrease in MHC I expression (Figure 3E). MHC II expression on tumor cells was similar in WT and KO mice and not affected by tamoxifen or abemaciclib therapy. However, HTM in all groups displayed a broad range of MHC II expression levels (Figure 3E).

To assess the effects of treatment on metastasis capacity, the presence of tumor cells in the lung or the BM was evaluated by flow cytometry (Figure 3F). In general, the presence of disseminated tumor cells in these organs was, with very few exceptions, very low. At the end of the five-week treatment period, no significant changes in the percentage of metastasized/disseminated tumor cells in the lung or in the BM were detectable upon HER4 KO or tamoxifen or abemaciclib therapy (Figure 3F). Spleen weight tended to be reduced in WT mice in response to tamoxifen and abemaciclib, and it was significantly reduced in abemaciclib-treated KO mice (Figure 3G).

### 2.5. Impact of Tamoxifen and Abemaciclib on Immune Cell Activation and Infiltration

The immune cell composition in the spleen (that reflects treatment-related systemic effects) and in the tumor was determined to assess the immune response in tamoxifen- and abemaciclib-treated mice as a function of HER4. It is important to mention that the therapy was very effective in some mice and led to an almost complete elimination of the tumors. Accordingly, these tumors could not be examined for immune cell infiltration at the end of the experiments.

**Figure 4:**
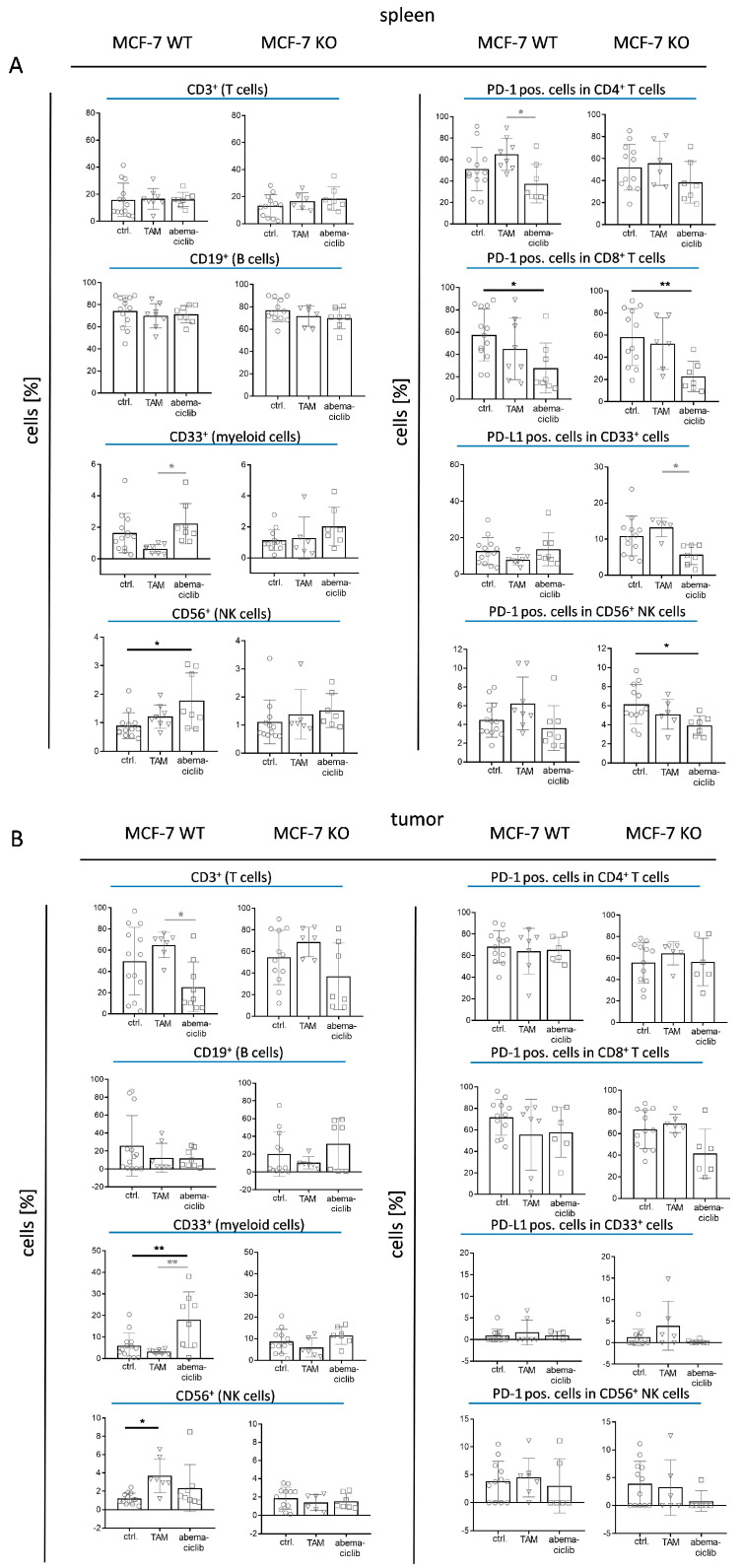
Immune cell distribution in the spleen and tumor of MCF-7-based humanized tumor mice (HTM). Flow cytometric analyses of (**A**) spleen and (**B**) tumors of HTM transplanted with MCF-7 WT or KO cells are shown. Each graph represents data from untreated (ctrl.), tamoxifen (TAM)-treated, or abemaciclib-treated mice. Gating was performed as described in Supplementary Figure S4 and the percentage of T (CD3), B (CD19), myeloid (CD33), and NK (CD56) cells of human CD45+ cells are displayed (left column). Percentage of PD-1 or PD-L1 for each population is displayed in the right column. Data are displayed as mean ± SD. Each mouse is represented by a single symbol and significances were calculated using one-way ANOVA and Tukey’s multiple comparisons test. Statistical significances: * *p* ≤ 0.05, ** *p* ≤ 0.01.

Immune cell infiltration of the spleen revealed a typical immune cell distribution pattern in young humanized mice (Figure 4A). B cells represented the most dominating immune cell population in WT and KO mice, at around 75% regardless of treatment. T cells represent 15% of the immune cells in the spleen and the distribution of CD4 and CD8 cells was very similar across all treatment groups with a ratio ~1. Interestingly, abemaciclib treatment caused a decreased PD-1 expression on CD4 and CD8 cells, which was significant for CD8 cells in WT and KO mice. Tamoxifen treatment slightly enhanced PD-1 expression on CD4 T cells in WT mice, which was significantly different from the checkpoint phenotype under abemaciclib treatment. No significant effect of abemaciclib or tamoxifen treatment on the proportion or PD-L1 expression of CD33 myeloid cells was seen compared to controls. NK cells, which were present at very low levels in the spleen, were increased upon treatment with abemaciclib in WT mice, but with a tendency of reduced PD-1 expression in WT and a significant reduction in KO mice.

As shown before in MCF-7 transplanted HTM, immune cell infiltration in the tumor tissue was low irrespective of treatments (MCF-7 WT ctrl: 2.76 ± 4.23; MCF-7 KO ctrl: 3.47 ± 5.40) and regardless of the HER4 receptor status (WT vs. KO). In untreated mice, T cell infiltration was highly variable between individuals, but tamoxifen treatment induced a cluster formation towards increased T cell infiltration, whereas abemaciclib decreased the infiltration capacity (Figure 4B). The CD4/CD8 ratio, which was ~1.1 in the spleen, increased towards a ratio of about 4.0 in the tumor. The PD-1 expression on CD4 and CD8 T cells infiltrating the tumor tissue was increased compared to those isolated from the spleen but without significant changes caused by treatments.

The WT mice treated with abemaciclib showed an increased infiltration of myeloid cells into the tumor without any changes in PD-L1 expression (Figure 4B). Tamoxifen treatment had no impact on myeloid cells, but an elevated level of NK cells was found in WT mice. However, it must be noticed that the total level of NK cells and their PD-1 expression level was generally low.

### 2.6. Cytogenetic Analyses of mdm2/CEN12 in Tumor Tissues Derived from Abemaciclib-Treated Patients

**Figure 5:**
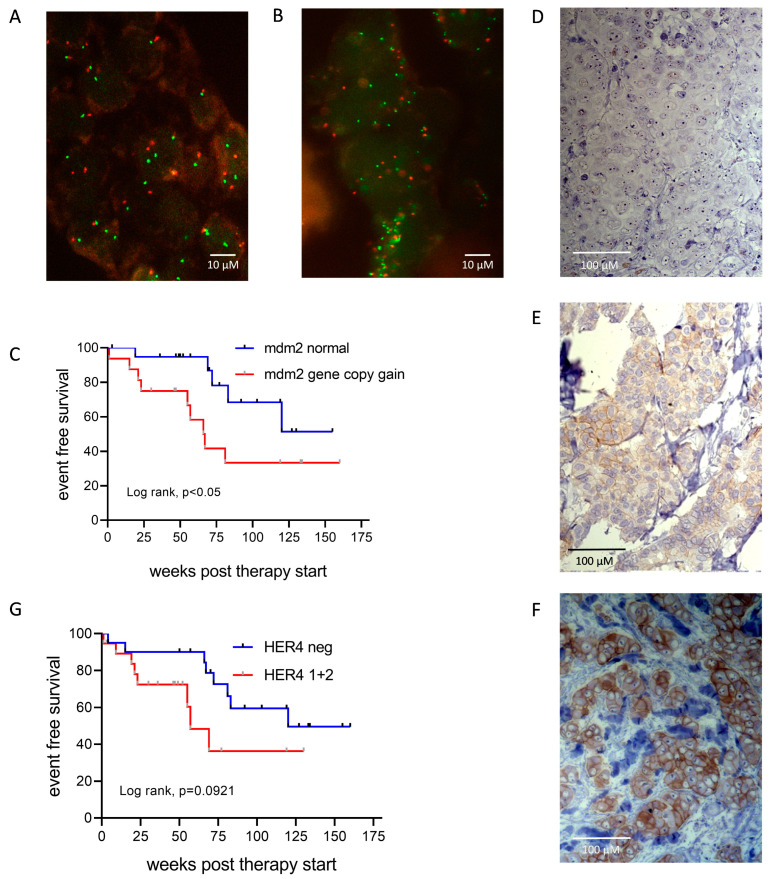
Event-free survival as a function mdm2 gene copy numbers and HER4 expression. Representative FISH images of mdm2 (green dots) and CEN12 (red dots) without (**A**) and with (**B**) signal gains are shown. (**C**) Kaplan–Meier graph illustrating EFS of luminal BC patients treated with abemaciclib as a function of genomic mdm2 and CEN12. Statistical differences were analyzed by Log-rank (Mantel–Cox) test. (**D**–**F**) Immunohistochemical HER4 stainings scoring “0” (no expression, “1” (low expression), and “2” (pronounced expression) are exemplified, respectively. (**G**) Kaplan–Meier graphs illustrating EFS of luminal BC patients treated with abemaciclib as a function of HER4 expression. Log-rank (Mantel–Cox) test.

A total of 39 tissue samples were evaluated with respect to the genomic mdm2 and CEN12 status (Supplementary Table S1). Compared to the signal evaluation in non-malignant breast epithelia, the lower and upper threshold for signal losses or gains (incl. ± SD) was set at 1.6 and 2.4 (CEN12) 0.8 and 1.2 (mdm2), respectively. Based on these categories, no real mdm2 gene amplifications were identified (i.e., elevated mdm2/CEN12 ratios). Instead, we found 16 samples with simultaneous gains in mdm2 and CEN12 signals, indicating a moderate chromosome 12 polysomy and a corresponding increased mdm2 gene dose. In contrast, 21 samples did not bear mdm2 or CEN12 signal alterations. FISH examples with and without alterations are shown in Figure 5A,B. Appropriate patient dichotomization revealed that patients harboring tumors with CEN12 polysomy/pronounced mdm2 signals showed a significantly (*p* = 0.049) shorter event-free survival (EFS) compared to patients suffering from tumors without an mdm2/CEN12 gain (Figure 5C). The maximum observation time was 160 weeks post-diagnosis.

### 2.7. HER4 Receptor Expression in Tumor Tissues Derived from Abemaciclib-Treated Patients

A total of 39 tumor samples could be included in the analysis. HER4 IHC staining patterns and intensities were categorized into a scoring system and given a score of “0” (HER4 negative), “1” (moderate staining intensity and/or heterogeneous HER4 expression), or “2” (strong staining intensity of all tumor cells) (Supplementary Table S1). Examples for all three categories are given in Figure 5D–F. We found a trend (*p* = 0.0921) of a shorter EFS for patients with HER4 positive tumors (score “0” vs. score “1” or “2”) (Figure 5G).

## 3. Discussion

Next to long-established endocrine therapy options (based, e.g., on ESR or aromatase inhibitors) the CDK4/6 targeting, primarily with abemaciclib, became an additional mainstay for the treatment of luminal BC. However, acquired resistance to CDK4/6i is a very frequent phenomenon [41,42]. Hence, biomarkers and molecular mechanisms with an impact on treatment efficiency need to be identified. Due to its pronounced expression in luminal BC and its versatile actions, HER4 represents one promising candidate that could serve as a predictive marker in different treatment settings. The HER4-triggered downstream signaling involves a proven (namely, CDK4/6) and a potential (namely, mdm2) molecular target for the treatment of luminal BC [36,43,44]. Here, we investigated the effect of ESR, CDK4/6, and mdm2 targeting in the presence and absence of HER4 expression in three well characterized ESR-positive BC cells (i.e., MCF-7, T-47D, ZR-75-1).

### 3.1. Dynamic Proliferation Analyses of HER4 WT and HER4 KO Cells as a Function of Treatments

The valid verification of HER4 knockout in MCF-7, T-47D, and ZR-75-1 cells allowed the systematic comparative treatment analyses of these ESR-positive BC cells as function of HER4. Extensive proliferation analyses based on continuous cell labeling with BrdU and subsequent double staining with Hoe33258 and PI revealed an enhanced sensitivity of MCF-7 cells upon HER4 knockout to separately applied tamoxifen, abemaciclib, and AMG232 treatments. The enhanced or newly generated sensitivity to these treatments was mainly due to an additional cell cycle arrest in the G2/M-phase that was not seen in HER4 WT cells. Combined abemaciclib/AMG232 and tamoxifen/NRG1 treatments were most efficient, while the exposure to abemaciclib/AMG232 causes an otherwise not seen S-phase arrest and the simultaneous tamoxifen/NRG1 administration stops cells cycle progress rigorously and immediately in both the G1- and G2/M-phases.

Interestingly, the HER4 elimination by CRISPR/Cas9 knockout enhances the sensitivity of MCF-7 cells to all treatment modalities and even converts the mitogenic effect of NRG1 to inhibition. Because NRG1 shows a binding affinity only to HER4 and HER3, the reversed effect must be enabled by the exclusive binding of NRG1 to HER3 in the absence of HER4 in knockout cells [45,46]. In addition, a pronounced HER3–HER2 interaction takes place when HER4 KO cells are exposed to NRG1 (Figure 2E). This is, on the one hand, somewhat surprising because HER3, even though kinase defective itself, has been frequently described as a stimulator of proliferation, particularly when dimerized with HER2 [47]. On the other hand, HER3/HER4-specific ligands (heregulins/neuregulins) have been found to induce cell differentiation [48,49] and even apoptosis [48,49,50,51,52], thus eliciting tumor-suppressive effects. Different effects of HER3/HER4 ligands, however, seem to be cell type-specific and—even more important—obviously depend on the type of HER-receptors co-expressed with HER4. (Potentially, less defined environmental factors might play an additional role.) The way in which the exclusive NRG1/HER3 binding in KO cells and the pronounced HER2–HER3 interaction are causally connected to the considerably reduced cell proliferation needs to be explored in more detail (e.g., downstream signaling).

T-47D cells turned out to be highly sensitive to abemaciclib but only modestly sensitive to tamoxifen treatment. Exposure to NRG1 resulted in an accelerated cell proliferation in both HER4 WT and HER4 KO cells. T-47D cells are completely resistant to AMG232 treatment, which is doubtlessly due to a loss of p53 function in this cell line [38,39]. Accordingly, T-47D cell proliferation is independent of p53 and, thus, also independent of mdm2. We show here that the second most common mutation seen in luminal BC (i.e., p53 mutations [53]) is likely to curb other molecule-specific treatments, e.g., an anti-CDK4/6 or mdm2-targeting treatment.

ZR-75-1 WT and ZR-75-1 KO cells arrested in the G1-phase when exposed to abemaciclib and are even more responsive to AMG232 (G1- and G2/M-phase arrest), which cannot be further enhanced by a combined abemaciclib/AMG2132 administration. Notably, ZR-75-1 cells do not respond to tamoxifen or NRG1 treatment, either when applied alone or in combination. All treatments effects were not modified in ZR-75-1 upon HER4 knockout. Consequently, a direct effect of ESR on sensitivity to tamoxifen therapy can also be ruled out in this cell line, as the expression of ESR was slightly reduced as a result of HER4 knockout (Supplementary Figure S1). It is known that there may be a co-regulation of expression between HER4 and ESR, but further analysis is needed to investigate the exact impact of HER4 knockout on ESR expression in ZR-75-1.

By comparing the effects on all three cell lines, it becomes obvious that the response to the respective treatment modalities varies. Different treatment effects might be associated with different cell-specific molecular contexts [38,39] (and www.cellosaurus.org, assessed on 29 April 2024). More specifically, T-47D cells are known to bear not only p53 mutations but also a heterozygous PIK3CA mutation. A homozygous loss of the tumor-suppressor CDKN2A (p16) and a TAGA3-heterozygous PIK3CA mutation are characteristic for MCF-7 cells, while a homozygous loss of PTEN and HRAS is seen in ZR-75-1 cells. These genomic alterations might affect diverse treatment modalities, amongst them, abemaciclib and tamoxifen treatments, as analyzed in this study. Despite the different effects of HER4 knockout in three luminal cell lines, abemaciclib treatment proved to be most efficient in all three ESR-positive cell lines and seems to be superior over tamoxifen for the treatment of ESR-positive BC. Prospectively, the markers and mechanisms that modulate the effects of HER4 in different molecular contexts need to be identified.

### 3.2. Potential Interaction of HER4/4ICD and ESR/4ICD as a Function of Treatments

In order to evaluate a potential interaction between HER4/4ICD and ESR under well-defined conditions, we treated MCF-7 WT cells with tamoxifen, abemaciclib, E2, and NRG1, respectively. The Western blots in Figure 2C revealed a translocation of the ESR into the cell nucleus upon E2 treatment. ESR dimerization, induced by the specific ESR ligand E2 and followed by the nuclear translocation, is basically a known scenario [14,54]. Interestingly, not only the ESR but also the cleaved HER4 (i.e., 4ICD) was slightly upregulated and translocated towards the nucleus under the same treatment conditions. This observation is compatible with a previous report in which this common translocation has been associated with a growth-simulating effect in luminal BC cells [14] triggered by the 4ICD-induced coactivation of the ESR [9,14,55]. This coactivation additionally drives the HER4 receptor expression itself via an autocrine signaling loop [14]. However, immunoprecipitation data revealed a HER4/4ICD–ESR interaction under untreated conditions but less interaction upon E2 treatment. Thus, we suggest that the role of 4ICD as an ESR co-activator in the absence of treatments may be secondary in the presence of high estrogen levels. Tamoxifen, which also binds to the ESR but with less affinity, leads to a moderate upregulation of ESR and HER4/4ICD levels (potentially a treatment-compensating effect) and does not impair ESR/4ICD interaction (Figure 2C,D). This finding is in agreement with our previous study, which showed an unfavorable course of disease for patients with HER4(4ICD)-positive, tamoxifen-treated, luminal BC [18]. Accordingly, we previously suggested an insufficient response of HER4-positive BC to tamoxifen treatment and consistently show here an enhanced sensitivity of MCF-7 HER4 KO cells in vitro. The HER4 knockout-related sensitivity is impressively evident in both short-term (Figure 1B) and long-term settings (Figure 2A). Moreover, the HER4/4ICD interaction was slightly elevated in the presence of abemaciclib and is compatible with less sensitivity of MCF-7 WT cells compared to HER4 KO cells (Figure 1B and Figure 2B,D), in which an ESR/4ICD interaction cannot occur.

In the presence of NRG1, a reduced 4ICD/ESR-complex formation is obvious, which is probably due to the downregulation of HER4/4ICD (Figure 2C). Last but not least, we identified only a moderate HER2–HER3 interaction in MCF-7 WT but a pronounced interaction of these two receptors in MCF-7 KO cells, when exposed to NRG1 (Figure 2E). In the absence of HER4, the HER3 receptor seems to preferably dimerize with HER2 upon the NRG1 binding that goes along with an inhibited cell proliferation of MCF-7 KO cells (Figure 1B). This is obviously the result of a modified HER3-triggered receptor signaling in the absence of HER4

Based on the data given in Figure 2C–E, we postulate a model that can explain how HER4/4ICD affects tamoxifen and abemaciclib treatment efficiency (Figure 2F). Upon release into the intracellular compartment, the 4ICD competes with tamoxifen for ESR binding and, thus, reduces the inhibitory effect expected from tamoxifen treatment. Accordingly, an HER4 knockout enhances the sensitivity to the estrogen antagonist tamoxifen. Cyclin D represents a key target gene of the estrogen receptor. ESR stimulation/activation by 4ICD binding causes pronounced Cyclin D expression, which, in turn, reduces the treatment effect of abemaciclib.

### 3.3. Growth of MCF-7 WT and MCF-7 KO Tumors in HTM Untreated and Treated with Abemaciclib or Tamoxifen

HTM bearing MCF-7 WT and MCF-7 KO tumors were treated over a period of 35 days either with tamoxifen or abemaciclib. In accordance with other studies, we administered tamoxifen and abemaciclib as mono-treatments, respectively [56]. This approach allowed deciphering the drug-specific effects in vivo. We observed a decelerated tumor growth in mice transplanted with MCF-7 WT cells and, inversely, an accelerated growth of MCF-7 tumors upon HER4 knockout in MCF-7 HTM. This might be due to two possible reasons: Either the HER4 receptor might elicit some kind of growth inhibiting effect, or there is a curbed stimulating effect in the presence of HER4 that is reactivated in the absence of HER4. Both potential effects might be caused by receptor-specific ligand binding [45]. For instance, the epidermal growth factor is known to elicit mitogenic activity upon binding to the EGFR; however, this receptor activation might be more efficient when an EGFR/HER4 interaction cannot occur in the absence of HER4 [57]. Additionally, the upregulation of HER2 receptor expression in MCF-7 KO mice might contribute to this effect. Alternatively, an active HER4 might tend to cause anti-proliferative and pro-differentiation effects in HTM, which cannot take place in tumors with HER4 knockout. The molecular mechanisms that drive cell and tissue differentiation via HER4 have been frequently attributed to the HER4 receptor but might not necessarily occur upon ligand binding and may also take place ligand-independently [3]. Moreover, a pro-apoptotic action of HER4/4ICD with Bcl-2 homology (a so-called BH3-only protein) can play a role in HER4 WT tumors [8,9]. These considerations are compatible with many studies in which a favorable prognosis has been attributed to HER4-positive BC [19,20,58,59,60,61]. Nevertheless, analyses of the preferred intracellular localization (cytoplasmic vs. nuclear) of HER/4ICD could provide helpful insight [10,62,63].

Both the abemaciclib and tamoxifen treatments efficiently inhibit the growth of MCF-7 WT tumors (no increase of tumor volume or weight). In MCF-7 KO HTM, both treatments show equivalent treatment efficiencies as well; however, a marginal tumor growth remained in the tamoxifen-treated mice. The very slight difference observed in treated WT and KO HTM can be attributed to the initially faster tumor growth of HER4 KO tumors. Thus, an enhanced therapy efficiency upon HER4 knockdown could not be observed in this study, because the WT cells appeared highly sensitive ab initio; therefore, this HTM-based study reflects a prognostic impact rather than a predictive effect of HER4, at least when highly sensitive tumor cells are used. However, it must be considered that the treatment period monitored in this study is significantly shorter than the therapy range used for patients. Tumor progression and the development of therapy resistance typically takes much longer, particularly in luminal BC [64,65]. Considering HER4-dependent tumor growth in HTM, the observation time of tumor growth in HTM is relatively short (~five weeks) and only partially reflects a long-time disease (i.e., progression). Thus, the total observation time applied in this study was based on existing directives for animal studies but insofar as human patients are concerned, it represents a limitation.

### 3.4. Potential Impact of Tamoxifen and Abemaciclib on Immune Cell Activation and Infiltration

To our knowledge, this is the first HTM-based study that addressed the (potential) immunological effects of tamoxifen and abemaciclib treatments. We could not identify any impact of tamoxifen treatment on the immune status in MCF-7 WT or KO-transplanted humanized mice. However, the abemaciclib treatment induced immunological changes, which point towards a, immunosuppressive reaction, especially in HER4 KO mice. Abemaciclib-treated HTM showed a reduced spleen weight (less proliferative activity) and reduced PD-1 expression on CD8^+^ T and NK cells (reduced activation). Notably, other researchers who used mouse BC models also reported a reduced expression of checkpoint molecules on immune cells upon CDK4/6 inhibitor therapy [66,67]; however, they associated this finding with less T cell exhaustion. Schaer et al. also described a reduced spleen cell count upon abemaciclib treatment in BALB/c mice, but supposed an association with reduced tumor burden [68]. In addition to a reduced PD-1 expression on splenic CD8^+^ T cells, the abemaciclib-treated WT HTM analyzed in this study showed a downregulation of MHC I on tumor cells. This MHC I downregulation has been described as an immune evasion mechanism associated with a decreased intratumoral immune cell infiltration and activity [69]. This possible negative impact of CDK4/6i on the adaptive immune response has been postulated previously, because the inhibitors also block cyclin D2- and cyclin D3-dependent G1-phase progression and, thereby, T-cell expansion [68]. Moreover, compared to KO HTM, abemaciclib-treated WT mice showed a significantly increased proportion of tumor-infiltrating CD33^+^ myeloid cells but with modest expression of the immunosuppressive ligand PD-L1. However, in a majority of publications, an immune-stimulating effect of CDK inhibition is suggested, for example, by a suppressed regulatory T cell proliferation [67] or an induced secretion of immune-stimulatory cytokines/chemokines [70]. Furthermore, a pronounced MHC expression and antigen presentation on immune cells and the induction of an inflamed tumor microenvironment has been reported [68]. In a murine syngeneic BC model, the combination with checkpoint therapy further intensified the T cell activation and the anti-tumor efficacy of abemaciclib. Different clinical trials for BC patients combining CDK4/6i and checkpoint inhibitors (i.e., PD-1, PD-L1) have been started but the initial results from these ongoing clinical trials do not show enhanced responses for this treatment compared to CDK4/6i monotherapy [71]. Therefore, extending studies in appropriate preclinical mouse models and in humans is needed to comprehensively understand the immune-modulating effect of CDK4/6i and to define the best combination therapies for BC patients.

### 3.5. Outcome of Abemaciclib-Treated Patients as a Function of Tumor-Associated mdm2 Gene Copy Numbers and HER4 Expression

We found an association between enhanced HER4 expression and shorter EFS for abemaciclib-treated patients. Presuming that HER4 expressed in luminal BC drives CDK4/6 signaling [1,36], it is not surprising that those tumors do not efficiently respond to abemaciclib treatment. Consequently, patients with HER4-positive tumors suffer from earlier disease progression and would potentially benefit from an additional targeting, for example, of mdm2 [72].

Mdm2 has been previously described as having an unfavorable effect on the course and outcome of tumor diseases (incl. BC) [73]. This can be explained by a pronounced degradation of p53 (and p21) that results in a reduced ability to control the (defective) genome and to guide cells towards apoptotic cell death [74]. Considering, exclusively, the luminal BC subtype, we previously reported in detail that mdm2 gene amplification is associated with progression and a worse outcome of disease [34]. Here, we observed a significant association between a shortened EFS of abemaciclib-treated patients with metastasized luminal BC and increased mdm2 gene copy numbers in tumor cells, even though the analyzed cohort is small yet. (The limited cohort size is due to the fact that abemaciclib therapy is not yet standard for years). Nevertheless, the finding suggests a connection between mdm2 and CDK4/6 signaling. A mechanistic model for protein and transcriptional interaction between the CDK4/6 and mdm2/p53 signaling axes has been proposed earlier [43]. Moreover, mdm2 downregulation/inactivation has been postulated as response mechanism for anti-CDK4/6 therapy. More specifically, CDK4/6 targeting (e.g., by abemaciclib) can drive tumor cells into proliferative quiescence (which is reversible); however, a reduced impact of mdm2 seems to be an essential mechanism for CDK4/6i-induced senescence, which is considered an irreversible precursor stage towards apoptosis [75,76]. Thus, CDK4/6i-treated cells with an active (or overexpressed) mdm2 can reside in the reversible quiescent state, thereby preserving their ability to revert to proliferation. This mechanism explains the rather poor response and outcome of abemaciclib-treated patients bearing tumors with pronounced mdm2 gene dosage and expression, as found in this study. Even though the analyzed patient cohort is still small, the impact of mdm2 (and HER4) becomes visible. Prospectively, it might be useful to verify a low myeloid cell infiltration and increased MHC I expression in HER4-negative tumors in patients, as seen in HTM. Overall, our findings require validation by additional advanced 3D preclinical [77] and clinical research.

## 4. Materials and Methods

### 4.1. BC Cell Lines and Treatments

All cell lines used in this study were authenticated by the German Collection of Microorganisms and Cell Cultures GmbH (DSMZ, Braunschweig, Germany). ER/HER4 double positive human BC cell lines MCF-7 (American Type Culture Collection no. HTB-22™, RRID:CVCL_0031), T-47D (ATCC no. HTB-133™, RRID: CVCL_0553) and ZR-75-1 (ATCC no. CRL-1500™, RRID:CVCL_0588) were cultivated in DMEM (MCF-7, T-47D) or RPMI (ZR-75-1) supplemented with 5% fetal bovine serum (Thermo Fisher Scientific, Waltham, MA, USA) at 37 °C and 5% CO_2_.

For short-term treatments, BC cell lines were treated with 10 nM 17-β-estradiol (Sigma-Aldrich, St. Louis, MO, USA), 5 µM (Z)-4-Hydroxytamoxifen (Sigma-Aldrich), 30 ng/mL NRG1 (Recombinant Human NRG1-beta 1/HRG1-beta 1 ECD Protein, R&D Systems, Minneapolis, MN, USA), 100 nM abemaciclib (Selleck Chemicals, Cologne, Germany), 100 nM AMG232 (Axon Medchem LLC, Reston, VA, USA) and various combinations thereof. To generate tamoxifen- and abemaciclib-resistant cell lines, the cells were continuously exposed to increasing concentrations of tamoxifen (from 10 to 4000 nM) or abemaciclib (from 1 to 100 nM) over a period of up to 300 days.

### 4.2. HER4 Receptor Knockout by CRISPR/Cas9 RNP Transfection and Clone Expansion—Verification of Stable HER4 Knockout (KO)

MCF-7, T-47D, and ZR-75-1 cells were transfected at 80% confluence with a ribonucleoprotein (RNP) complex consisting of the previously designed specific single guide RNA (Hs.Cas9.ERBB4.1.AD; CAATGTGACGGCAGATGCTA) and the Cas9 protein (both acquired from Integrated DNA technologies; IDT, Coralville, IA, USA). Single guide RNA was predesigned to bind in one of the first exons so that only a small part of the HER4 gene was translated. Nucleofection was performed using the Cell Line Nucleofector^®^ Kit V and the Amaxa Nucelofector I device and technology (Lonza, Basel, Switzerland) according to manufactures protocol. In brief, cells were trypsinized and washed twice with PBS and 1 × 10^6^ cells were subsequently resuspended in nucelofector solution. After adding the electroporation enhancer (IDT) and the RNP complex, the cell suspension was transferred to an electrically conductive cuvette and specific electroporation program was conducted (MCF-7: P-20; T-47D and ZR-75-1: X-05). After 48 h cultivation, the electroporated cells were harvested, centrifuged (90 *g* for 10 min), and diluted to a concentration of 10 cells/mL. To grow clones from a single cell, 100 µL of prepared cell suspension was applied per well of a 96-well plate. The plates were incubated under standard conditions, cell growth was monitored every three days, and 100 µL of fresh medium was supplied weekly. For the further transfer and propagation to 6 well plates and T25 tissue flasks, only wells containing single clusters were picked.

The successful generation of a homozygous HER4 gene knockout was verified in a three-stage procedure. First, propagated cell clones were analyzed by flow cytometry for HER4 surface expression by a standard staining procedure using a APC-conjugated anti-HER4 (R and D Systems Cat# FAB11311A, RRID:AB_2277999) and corresponding isotype control (BioLegend Cat# 400221, RRID:AB_2891178) antibody. Only clearly HER4-negative clones were further propagated for protein isolation and subsequent detection of HER4 expression by Western blot. Clones that did not show a positive signal in either FACS or Western blot were considered to be successful knockout clones. The final verification was made by Sanger sequencing. For this, DNA was isolated with Wizard^®^ Genomic DNA Purification Kit (Promega, Walldorf, Germany) according to manufactures protocol. The CRISPR Cas9 gene locus was amplified by PCR using specifically designed primers binding approximately 100 bp up- or downstream of the CRISPR locus (fw: ATAAGACAAAGATTCAGTATGCCTG, rv: GCTGAATTGAGTCAAAGACAGG). The PCR product was purified with Wizard^®^ SV Gel and PCR Clean-Up System (Promega) according to the protocol supplied. Finally, Sanger Sequencing was done to identify specific gene alterations (i.e., deletions, insertions) for each successfully generated KO clone by Thermo Fisher Scientific GENEART GmbH (Regensburg, Germany). Sequencing data were finally analyzed using Geneious Prime Software (V.2022.1, Biomatters, New Zealand).

### 4.3. Dynamic Proliferation Assessment by Flow Cytometry

Flow cytometric BrdU/Hoechst quenching measurements were performed as described previously in more detail [78,79,80,81]. In brief, cells seeded at appropriate cell densities were continuously exposed to 120 µM bromodeoxyuridine (BrdU) and 2’deoxycytidine at half-equimolar concentrations, which enabled the incorporation of BrdU (instead of thymidine) into the DNA. Cells were harvested in intervals covering a time range up to 96 h. After detachment, the cells were stored at −20 °C at a concentration of 10^6^ cells/mL in freezing medium (Rosewell Park Memorial Institute 1640 medium +10% FCS +10% dimethyl sulfoxide [DMSO]) until flow cytometric analysis. For cell staining, thawed cells were washed twice with 2 mL of ice-cold DNA-staining buffer (100 mM Tris-HCl, pH 7.4, 154 mM NaCl, 1 mM CaCl_2_, 0.5 mM MgCl_2_, 0.1% IGEPAL CA-630 [Nonylphenylpolyethyleneglycol], 0.2% BSA). 5 × 10^5^ cells were resuspended in 1 mL buffer supplemented with 40 g/mL (2–4 Units/mL) RNase and 1.2 μg/mL Hoechst 33258 (Sigma-Aldrich) and incubated for 15 min at 37 °C. Cellular DNA was additionally stained with propidium iodide (1.5 μg/mL) for 15 min on ice. Flow cytometric measurements were done on a FACSCanto-II (BD Biosciences, San Jose, CA, USA) equipped with three lasers (standard optical configuration). Sample measurements and data analysis were performed with FACSDiva Software v7.0 (BD Biosciences). A total of 50.000 events/sample were collected.

### 4.4. Characterization of Tumor and Immune Cells

The human reconstitution and the phenotyping of human immune and tumor cells were investigated by flow cytometry. Single cell suspensions from lung, tumor, and spleen were obtained by mincing the organs with PBS through a 40 µM cell strainer (Thermo Fisher Scientific). To collect the bone marrow (BM) cells, the femur was cut and the bone cavity flushed with PBS. Prior to staining, cells were incubated with 1% mouse serum in order to avoid unspecific bindings. Antibodies were incubated for 30 min at 4 °C and erythrocytes were lysed for 10 min using FACS lysing solution (BD Bioscience). Antibodies against the following antigens/markers and conjugated with following fluorochromes were used: CD45-BV510 (BioLegend Cat# 304036, HI30, RRID:AB_2561940), CD33-PerCP-Cy5.5 (BioLegend Cat# 303414, WM53, RRID:AB_2074241), CD19-PE (Thermo Fisher Scientific Cat# 12-0198-42, SJ25C1, RRID:AB_10734045) and CD4-APC-H7 (BD Biosciences Cat# 641398, SK3, RRID:AB_1645732), CD56-PeCy7 (BioLegend Cat# 362509, 51H11, RRID:AB_2563926), CD3 BD Biosciences Cat# 555332, UCHT1, RRID:AB_395739), PD-1-AF647 (BioLegend Cat# 329910, EH12.2H7, RRID:AB_940471), PD-L1-BV510 (BioLegend Cat# 329714, 29E2A3, RRID:AB_2563852), HER2 (BioLegend Cat# 324410, 24D2, RRID:AB_2099256), EpCAM-AF647 (BioLegend Cat# 324212, 9C4, RRID:AB_756086), MHC I-PE (Thermo Fisher Scientific Cat# MA1-10346, MEM-123, RRID:AB_11154825), MHC II-BB700 (BD Biosciences Cat# 742224, Tu39, RRID:AB_2871434). EpCAM-positive tumor cells were examined for the expression of MHC I, MHC II, HER2, and PD-L1. Different immune cell populations (i.e., myeloid cells, B cells, CD4+ T cells, CD8+ T cells, and NK cells) were gated from human CD45+ leukocytes and analyzed for PD-1 and PD-L1 expression. Gating was adjusted according to the respective isotype controls and is shown in Supplementary Figure S4.

### 4.5. Immunohistochemistry

After organ preparation, tissue sections of lung and tumor were fixed in 4% formalin and embedded in paraffin and then cut in 1.5-μm paraffin sections. Prior to antibody staining, the paraffin specimens were deparaffinized, heated up in Tris-EDTA buffer (pH = 9) to 120 °C and incubated for 5 min. After blocking the endogenous peroxidase with peroxidase blocking solution (Dako, Glostrup, Denmark), the slides were washed and primary antibodies were incubated for 30 min at RT. The following antibodies were used: anti-HER2 rabbit (Agilent Cat# A0485, dilution 1:500, RRID:AB_2335701) and anti-HER4 (Abcam Cat# ab32375, E200; dilution 1:20, RRID:AB_731579). The immunohistochemical staining was done automatically by Dako EnVision autostainer (HER2 staining) or manually (HER4 staining). Specific staining was detection based on horseradish peroxidase (HRP) reaction. Finally, counterstaining with hematoxylin was performed.

### 4.6. Protein Isolation and Western Blot

Total protein isolation was performed on ice using cell lysis buffer (Cell Signaling Technology, Danvers, MA, USA) supplemented with Halt™ Protease (Thermo Fisher Scientific) and Phosphatase Inhibitor Cocktail (Carl Roth, Karlsruhe, Germany). In order to separate the nuclear and cytoplasmic protein fractions, NE-PER Nuclear and Cytoplasmic Extraction Kit (Thermo Fisher Scientific) was used according to the protocol provided. The protein concentration was quantified using the Pierce BCA Protein Assay Kit (Thermo Fisher Scientific). According to protein size, 20 µg protein per lane was separated in 10 or 15% SDS-PAGE under reducing conditions (mercaptoethanol) and subsequently blotted onto polyvinylidene difluoride (PVDF) membranes. The membranes were then blocked for 1 h in TBS-T buffer 5% BSA or 5% low-fat milk and 1% Tween. Primary antibodies were incubated overnight in 5% BSA or 5% low-fat milk. The following antibodies were used and obtained from Cell Signaling Technology: p53 (Cat# 9282, RRID:AB_331476), MDM2 (Cat# 86934, RRID:AB_2784534), HER1 (Cat# 2232, RRID:AB_331707), HER4 (Cat# 4795, RRID:AB_2099883). Further primary antibodies were used: ESR alpha (Santa Cruz Biotechnology, Dallas, TX, USA, Cat# sc-73479, RRID:AB_1122656); HER3 (Santa Cruz Biotechnology Cat# sc-81455, RRID:AB_1121503), HER2 (Sigma-Aldrich Cat# OP-15), AIB1 (BD Biosciences Cat# 611104, RRID:AB_398417). According to the protein size of interest anti-β-actin (Sigma-Aldrich Cat# A2066, RRID:AB_476693; 1:20.000) or Rab11 (Cell Signaling Technology Cat# 3539, RRID:AB_2253210; 1:1000) was used as loading control. As protein size standard, PageRuler plus prestained protein ladder (Thermo Fisher Scientific) was used. After washing the membrane, secondary antibodies, anti-rabbit (Cat# 7074, RRID:AB_2099233; 1:2000) or anti-mouse (Cat# 7076, RRID:AB_330924), from Cell Signaling Technology were incubated for 1 h at room temperature. Finally, visualization of the proteins was performed with the chemiluminescent substrate SuperSignal west pico PLUS (Thermo Fisher Scientific) and ChemiDoc Imaging System (Image Lab 6.0.1, BioRad, RRID:SCR_014210).

### 4.7. Immunoprecipitation

Co-immunoprecipitation (Co-IP) was performed using magnetic-activated cell sorting (MACS) technology adapted to the manufacturer’s instructions. 50 µL μMACS Protein A MicroBeads (Miltenyi Biotec, Bergisch Gladbach, Germany) and 200 µg protein lysate were mixed. Then, catcher antibody anti-HER4 (Cell Signaling Technology Cat# 4795, RRID:AB_2099883, 1:50) or anti-HER2 (Cell Signaling Technology Cat# 2165, RRID:AB_10692490, 1:50) was incubated for 6 h at 4 °C. μColumns (Miltenyi Biotec) were inserted into the magnetic thermoMACS separator (Miltenyi Biotec). After equilibration, the lysate mixture was transferred onto the column. While the magnetic beads were retained by the column, unbound proteins were removed in several washing steps with 200 µL ice cold lysis buffer. Finally, proteins bound to the magnetic beads were eluted with 50 µL Lämmli buffer (Carl Roth) under reducing conditions (mercaptoethanol) and denatured for 5 min at 95 °C. 10 µL of each sample was taken for Western blot analysis, using anti-ESR (Cell Signaling Technology Cat# 13258, RRID:AB_2632959) or anti-HER3 (Santa Cruz Biotechnology Cat# sc-81455, RRID:AB_1121503) detection antibodies.

### 4.8. Generation and Treatment of Humanized Tumor Mice (HTM)

HTM were generated as outlined previously [34,40,82,83] and illustrated in Supplementary Figure S4A. In order to humanize mice, CD34^+^ hematopoietic stem cells (HSCs) were isolated from umbilical cord blood. Following a Pancoll (PAN Biotech, Aidenbach, Germany) density gradient centrifugation (600 *g*, 30 min, room temperature) to separate mononuclear cells, CD34^+^ stem cells were isolated using immunomagnetic beads (Miltenyi Biotec RRID:AB_2848167) as described in the manufacturer’s protocol.

HSC quality and purity control was done by flow cytometry using PE-anti-humanCD34 (BioLegend Cat# 343505, RRID:AB_1731937) and αCD3-FITC (BD Biosciences Cat# 555332, RRID:AB_395739) antibodies. Female NOD.Cg-*Prkdc^scid^ Il2rg^tm1Wjl^*/SzJ mice (NSG; RRID:IMSR_JAX:005557) were irradiated with 1 Gy ~48 after birth, and 3 h afterwards, ~1 × 10^5^ HSCs were transplanted intrahepatically. Eight weeks later, the human reconstitution was verified in peripheral blood by flow cytometry using the antibodies against human CD45, CD33, CD19, and CD4 (detailed antibody information above). Successfully reconstituted (>20% human immune cells present in peripheral blood) females were transplanted with 6 × 10^6^ MCF-7 cells (diluted in 50 µL culture medium and mixed with 50 μL of Matrigel (R&D Systems; 3432-010-01) orthotopically in the mammary fad pad. Transplantation was performed under anesthesia (midazolam 5 mg/kg, fentanyl 0.05 mg/kg, and medetomidine 0.5 mg/kg i.p.) and shortly antagonized afterwards (flumazenil 0.5 mg/kg, atipamezol 2.5 mg/kg and naloxon 1.2 mg/kg). To enhance tumor cell engraftment and growth, mice received estrogen (8 µg/mL; Sigma-Aldrich, E2758) upon tumor transplantation diluted in the drinking water. HTM from the same cord blood donor were split equally between the control and treatment groups. Treatment started 12 days after tumor transplant when the tumors had a size of ~5 mm in diameter. Tamoxifen was administrated orally per tamoxifen citrate feeding pellets (400 mg/kg; sniff, A115T70404). Abemaciclib was dissolved in 25 mM buffer solution of phosphoric acid with 1% hydroxyethyl cellulose and pH = 2, diluted in raspberry syrup and administered orally for 5 days per week at a final treatment concentration of 50 mg/kg. Mice were monitored on a regular basis and tumor growth was measured twice per week and calculated according to following equation: tumor volume = ½ (length × width^2^). After 5 weeks of treatment, the mice were terminated and analyzed.

### 4.9. Fluorescence In Situ Hybridization (FISH) and HER4 Immunohistochemistry (IHC) Applied to Tumor Tissues Derived from Abemaciclib-Treated Patients

FISH on tumor tissue samples (n = 39) derived from patients treated with abemaciclib (Table 1) were hybridized with a SPEC MDM2/CEN12 dual color probe (ZytoVision GmbH, Bremerhaven, Germany). Tissue specimens were processed by the Institute of Pathology (University of Regensburg) as described previously [84]. The total hybridization procedure is described in detail by Holzschuh et al. as well [84]. Twenty-five non-overlapped single-cell nuclei were examined nucleus by nucleus by using an epifluorescence microscop (AxioImager-Z1; Zeiss, Göttingen, Germany). Two separate tissue areas from the same tissue sample (identified by a pathologist) were inspected. Mdm2 and CEN12 hybridization spots were independently quantified by two observers. Absolut signal counts and signal ratios were used for the analysis. Twenty non-tumor tissues derived from breast reductions were used to determine the level of unaltered mdm2 and CEN12 signals per cell. Based on control tissues, signal gains or losses were identified. Deviations from normal tissues with (±2 × SD) were considered to represent genomic alterations.

### 4.10. Statistical Analysis

The statistical analyses were done using GraphPad Prism (RRID:SCR_002798), version 6. Two-way ANOVA, *t*-test, or one-way ANOVA and either Dunnett’s multiple comparisons test or Tukey’s multiple comparisons test was performed as indicated in the figure legends. Kaplan–Maier curves were analyzed by Log-rank (Mantel–Cox) test. Differences with a *p*-value ≤ 0.05 were considered significant. Moreover, the significance was further classified depending on the strength of significance: not significant (ns) = *p* > 0.05; * *p* ≤ 0.05; ** *p* ≤ 0.01; *** *p* ≤ 0.001; **** *p* ≤ 0.0001. Data are presented as mean with standard deviation (SD) unless otherwise described.

## 5. Conclusions

HER4 differentially affects tamoxifen, abemaciclib, and AMG232 treatment efficiencies in MCF-7, T-47D, and ZR-75-1 ESR-positive BC cells in vitro. The cell line-specific differences must be due to the individual molecular contexts, not least, to the specific genomic mutations frequently seen in luminal BC (p53, PTEN, HRAS, PI3K). An HER4 knockout enhances the tamoxifen, abemaciclib, and AMG232 treatment efficiency in vitro, particularly in MCF-7 cells. Remarkably, the mitogenic effect of NRG1 is turned into inhibition of MCF-7 cell proliferation in this cell line. The treatment-independent tumor growth in vivo is accelerated upon HER4 knockout in MCF-7 based HTM. Nevertheless, abemaciclib and tamoxifen treatments are both efficient to curb tumor growth. Abemaciclib-treated patients suffering from luminal BC with HER4 expression or increased mdm2 gene copy numbers perform poorly compared to tumor patients without HER4 or mdm2 alterations. Thus, HER4 and mdm2, both putative key players for the CDK4/6 controlled cell proliferation (and survival), unfavorably affect the anti-CDK4/6 treatment. Extended preclinical treatment studies and prospective analyses of abemaciclib treatments as a function of HER4 and mdm2 are required for validation.

## Figures and Tables

**Table 1:** Patients’ demographic data. A total of 39 hormone receptor-positive patients were included into the analyses of mdm2/CEN12 FISH analysis and HER4 IHC. NST = invasive carcinoma of no special type; ET = endocrine therapy; NCT = neoadjuvant chemotherapy; Tx = chemotherapy and/or endocrine therapy.

Clinicopathological Parameters	Category	Size of Patient Cohorts (%)
age	≤50	10 (25.6%)
	>50	29 (74.4%)
histological type	NST	31 (79.5%)
	lobular invasive	7 (17.9%)
	other	1 (2.6%)
therapy ET + abemaciclib	ongoing	23 (58.9%)
	time on ET + abemaciclib (mean in months)	23.4
therapy ET + abemaciclib	progress or death	14 (35.9%)
	time on ET + abemaciclib	10.4
therapy ET + abemaciclib	first line	33 (84.6%)
	second line	4 (10.3%)
	never started	2 (5.1%)
metastasized/local advanced	after NCT	8 (20.5%)
	>5 years after 1. diagnosis	3 (7.7%)
	<5 years after 1. diagnosis	5 (12.8%)
	after adj. Tx	16 (41.0%)
	>5 years after 1. diagnosis	8 (20.5%)
	<5 years after 1. diagnosis	8 (20.5%)
	de novo	15 (38.5%)

## Data Availability

All data relevant to the study are included in the article or in the Supplementary Material.

## Supplementary Materials

The following supporting information can be downloaded at: https://www.mdpi.com/article/10.3390/ijms25137475/s1.
