# Supplementary material for: HER4 Affects Sensitivity to Tamoxifen and Abemaciclib in Luminal Breast Cancer Cells and Restricts Tumor Growth in MCF-7-Based Humanized Tumor Mice
Source: Int J Mol Sci. 2024 Jul 8;25(13):7475. doi: 10.3390/ijms25137475 (PMC11242770; doi:10.3390/ijms25137475)
Supplement: Supplementary file 1 [file ijms-25-07475-s001.zip › ijms-3054440-supplementary.pdf]

Supplementary Figures

Supplementary Figure 1. Generation and characterization of stable HER4 KO cells. (A) A timeline of CRISPR-/Cas9 gene HER4 gene knock-out is given. The ribonucleoprotein complex (consisting of the sgRNA and the Cas9 protein) was built and transfected into the cells by electroporation and cell clones were expanded and screened. Three independent approaches were used to verify HER4 gene knock-out in a number of clones of each cell line. (B) The absence of HER4 surface expression was analyzed by flow cytometry. In the given example for MCF-7, one clone (middle panel) appeared completely HER4 negative whereas an-other one (lower panel) shows just reduced HER4 expression compared to wildtype control cells (upper panel). (C) Protein lysates were generated from a number of expanded cell clones for western blotting. Again, one HER4 negative and one HER4 positive clone is shown. (D) Final verification was done by Sanger Sequencing after PCR based CRISPR-/Cas9 region-specific gene amplification. The example shows a deletion of six nucleotides (highlighted in red) nucleotide position 349.440 and 349.430 resulting in a stop-codon which is marked with “*” and labelled by black arrow. (E) One clone was selected per cell line and the protein expression of the related HER receptors and the ESR was investigated by western blot. (F) Doubling times of MCF-7, T-47D, ZR-75-1 cell lines and respective KO cell lines are given.

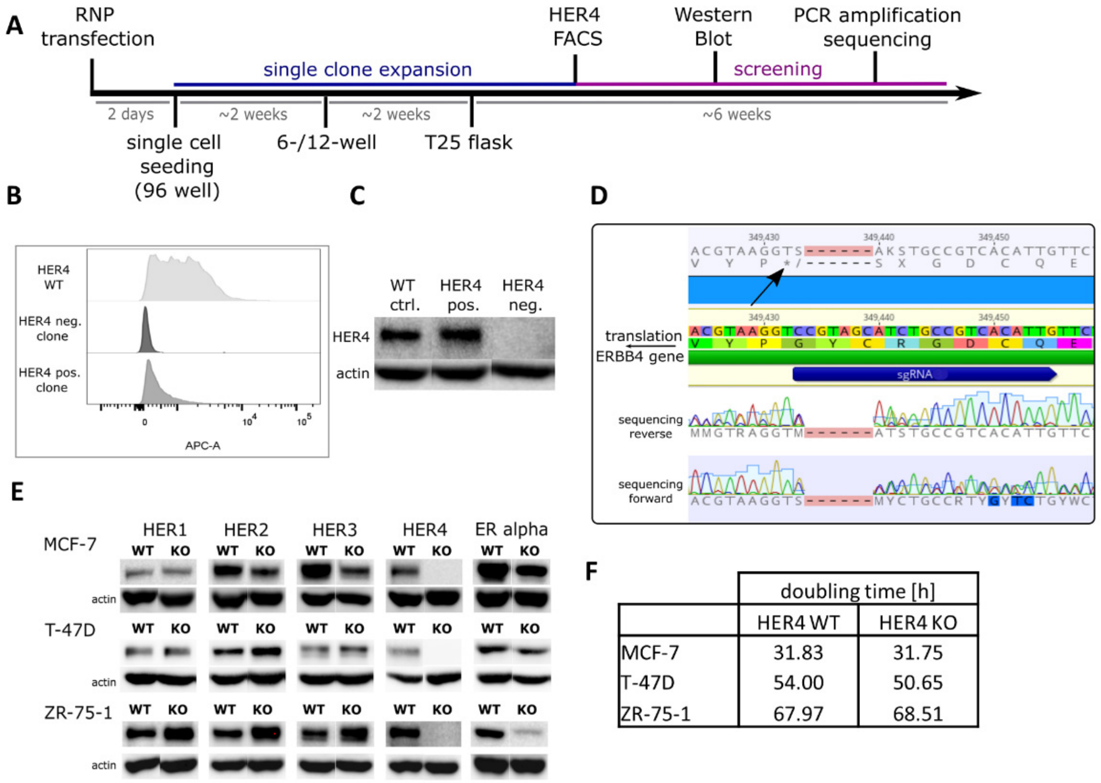

	doubling time [h]	
	HER4 WT	HER4 KO
MCF-7	31.83	31.75
T-47D	54.00	50.65
ZR-75-1	67.97	68.51

Supplement Figure 2: Cell cycle progression of (A) MCF-7, (B) T-47D, and (C) ZR-75-1 WT and KO cells as a function of treatments over a period of 96 h. Measurements were done in intervals of 8, 16, 24, 48, 72, and 96 h, respectively.

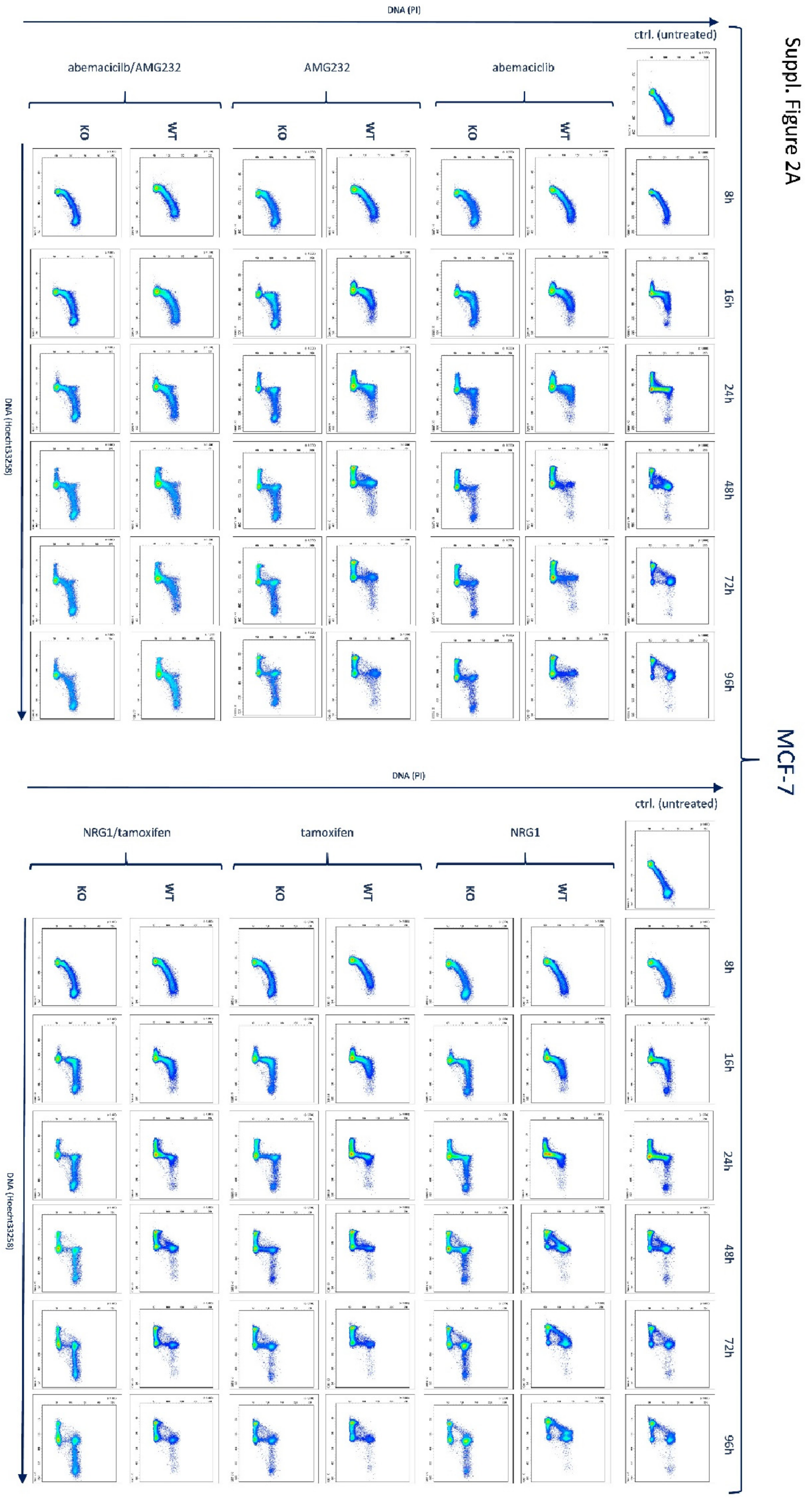

Suppl. Figure 2B

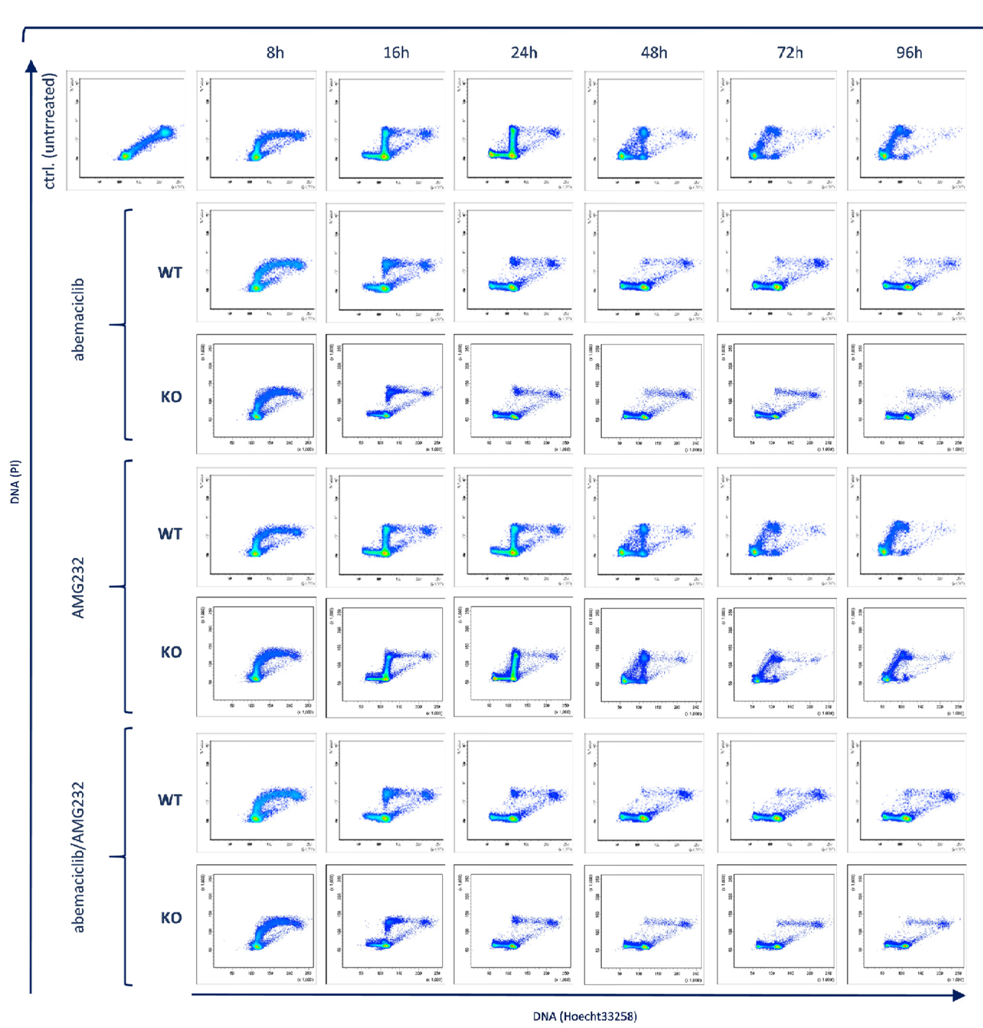

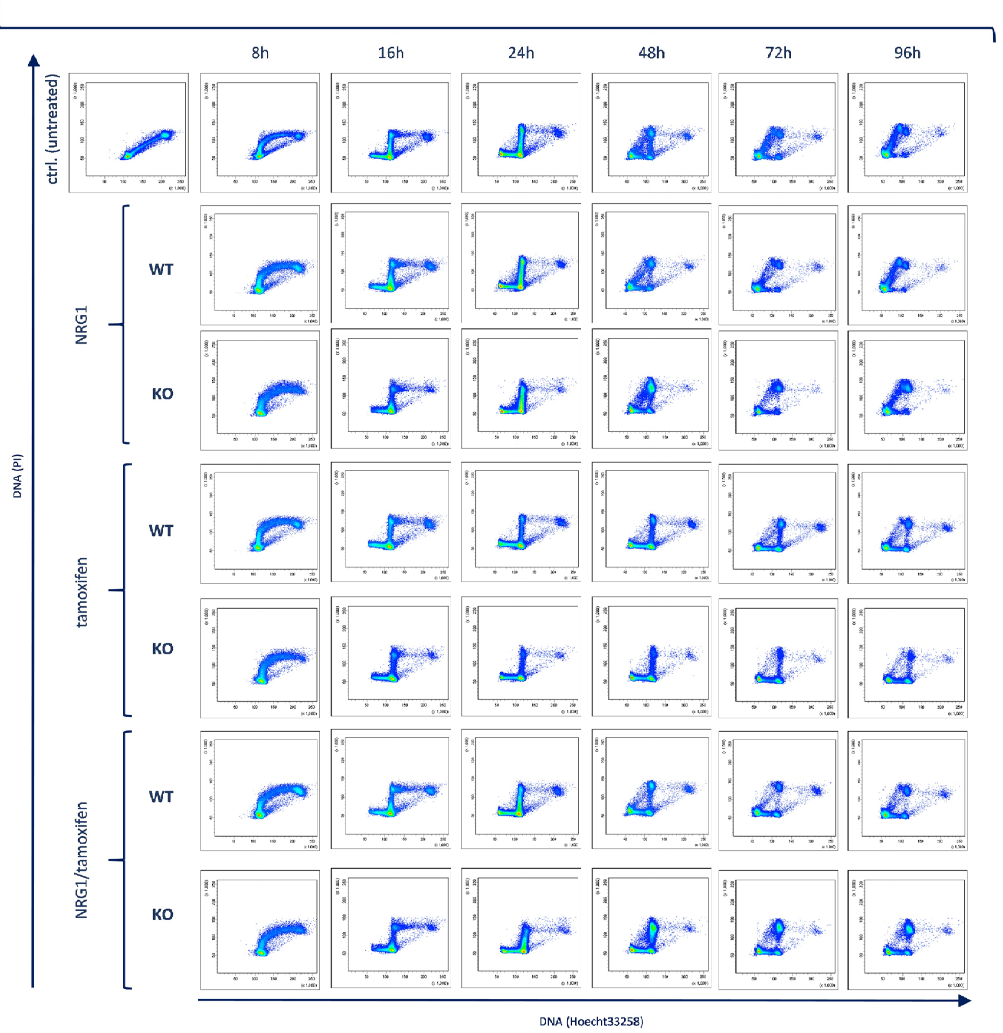

Suppl. Figure 2C

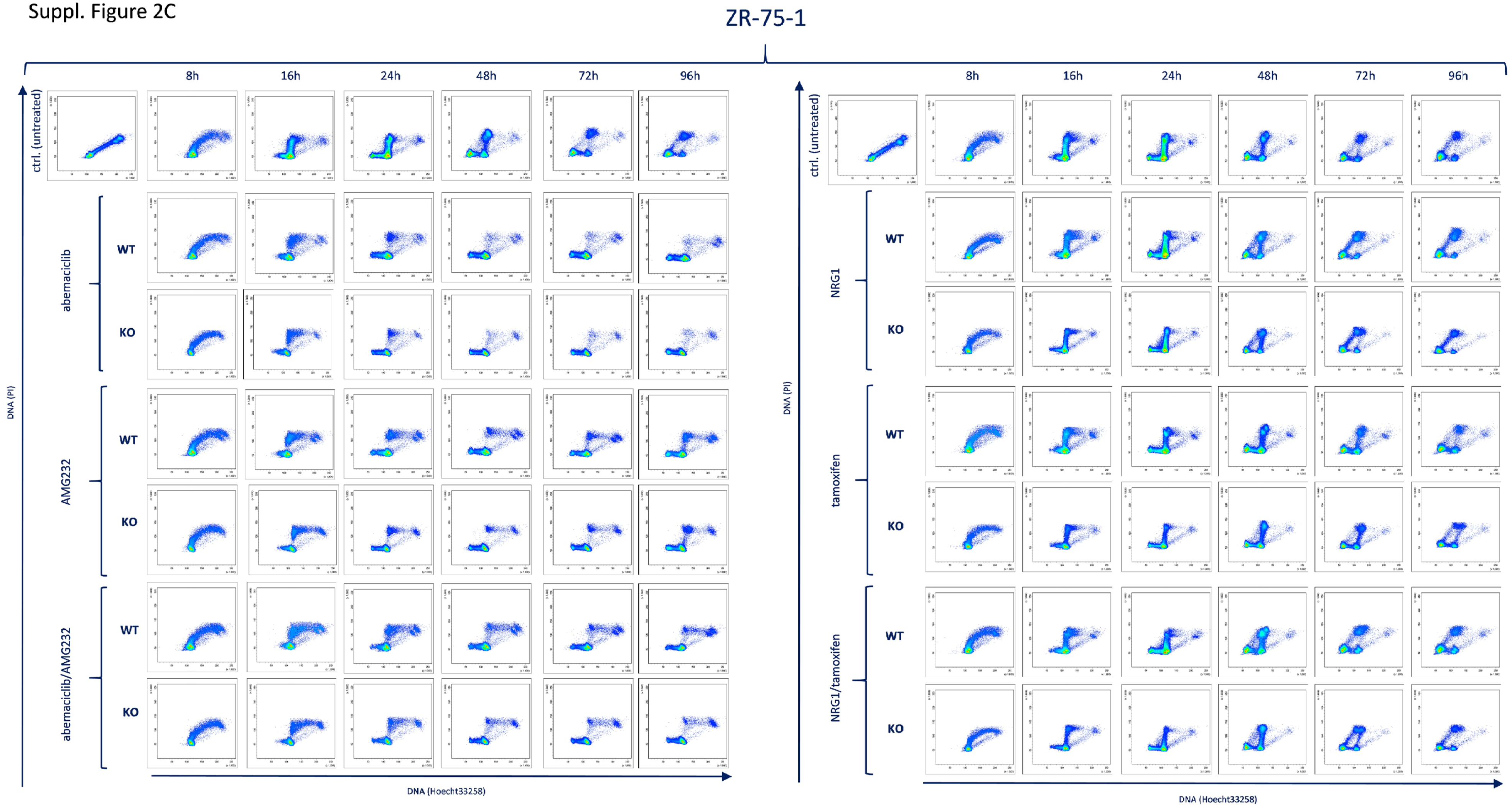

Supplement Figure 3: G1-phase (first cell cycle) exit curves of MCF-7, T-47D, and ZR-75-1 WT and KO cells. Percentages of cells attributed to the first cells cycle over a period of 96 h (x-axis) and as a function of treatments are displayed. The higher the value the more cells were prevented from progressing towards the second and third cell cycle. Cell cohorts within the first cell cycle were calculated as a subfraction of the total cohort (y-axis).

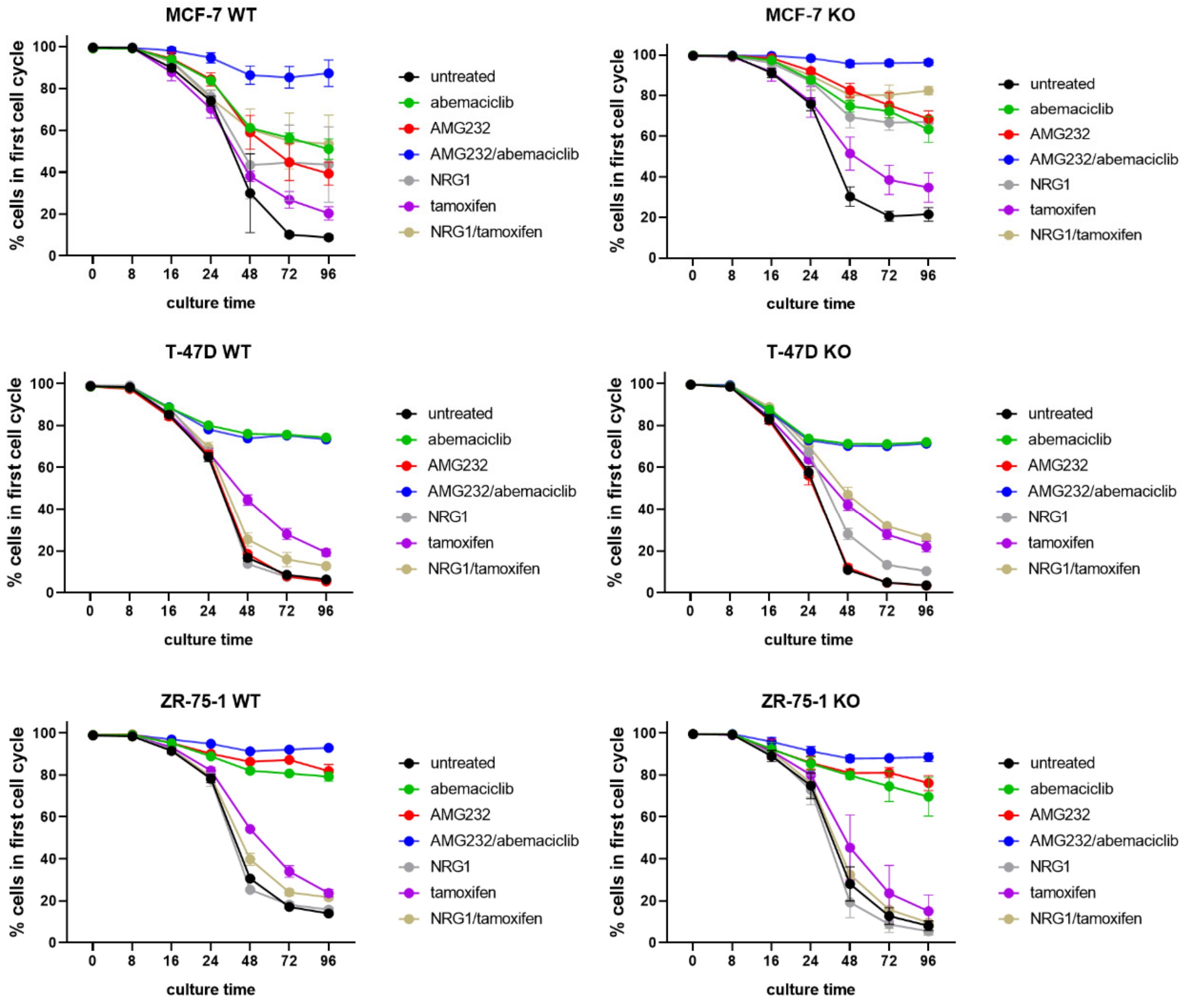

Supplement Figure 4: Phenotyping of immune and tumor cells by flow cytometry. (A) Time line of the mouse experiments is displayed. Successfully humanized (> 20% hCD45$^+$) were transplanted with 6 x 10^6 MCF-7 cells (i.e., WT or KO) and subsequently treated with tamoxifen or abemaciclib, respectively (B) Gating strategy is displayed for immune cell phenotyping. Different immune cell populations (i.e., myeloid cells, B cells, CD4$^+$ T cells, CD8$^+$ T cells and NK cells) were gated from human CD45$^+$ leucocytes and analyzed for PD-1 and PD-L1 expression. (C) EpCAM positive tumor cells were investigated for MHC I, MHC II, HER2 and PD-L1 expression. The gating was adjusted according to corresponding isotype controls.

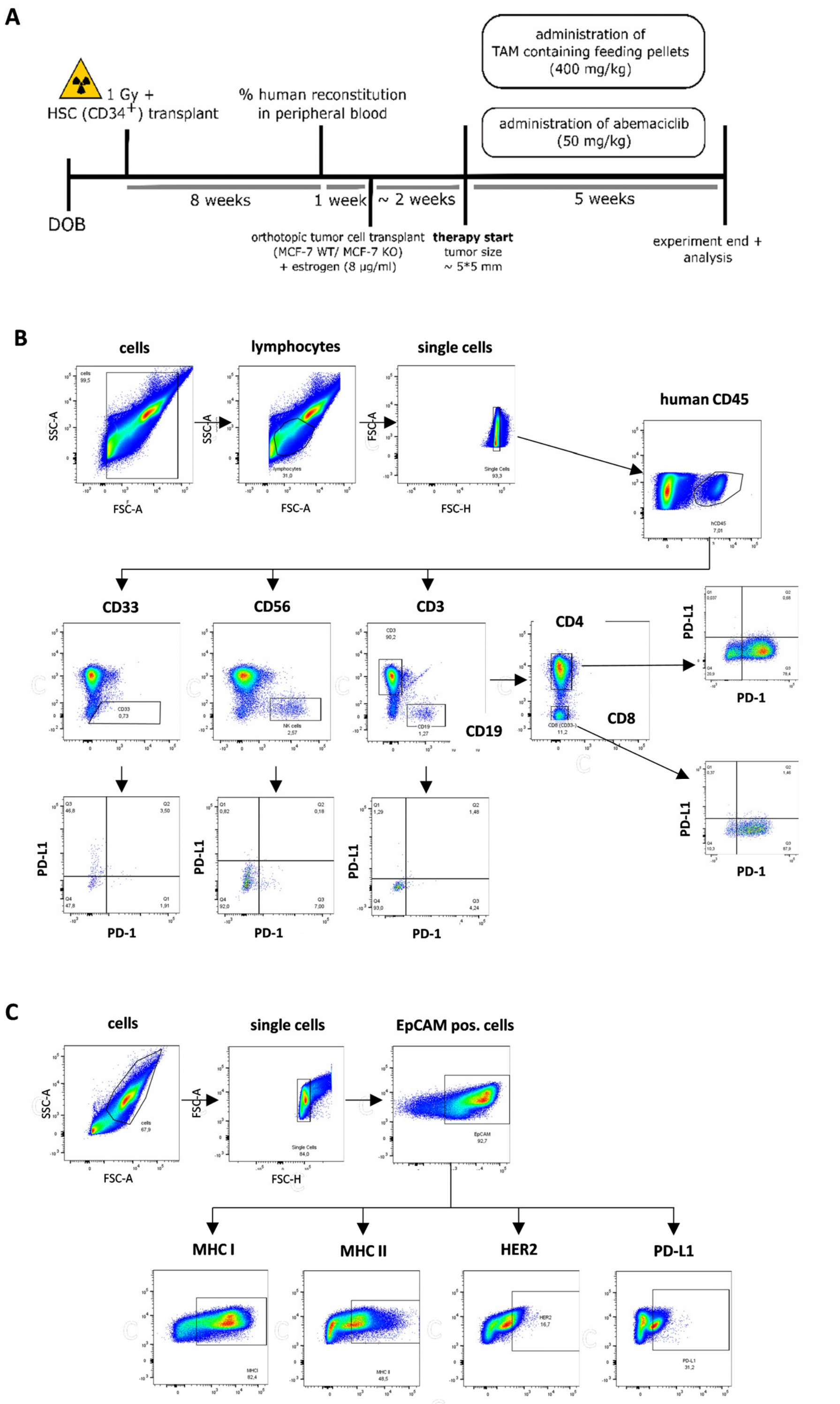

Supplementary Table 1

Patient #	hybridization signals / nucleus		mdm2 / CEN12 signal ratios	HER4 IHC score
	MDM2	CEN12		
1	2.39	2.29	1.04	0
2	n.a.	n.a.	n.a.	0
3	1.62	1.66	0.98	1
4	2.41	2.26	1.07	1
5	2.41	2.03	1.19	1
6	3.60	2.52	1.43	1
7	2.01	1.91	1.05	0
8	1.90	1.85	1.03	0
9	2.71	2.55	1.06	0
10	2.44	2.45	0.99	0
11	2.29	2.11	1.09	0
12	2.17	2.05	1.06	0
13	n.a.	n.a.	n.a.	2
14	2.52	2.36	1.07	0
15	1.85	1.83	1.01	2
16	2.16	2.09	1.03	2
17	3.47	3.17	1.09	2
18	1.7	1.76	0.97	0
19	2.21	2.17	1.02	0
20	2.44	2.92	0.84	1
21	2.43	2.59	0.94	1
22	2.95	2.66	1.11	1
23	1.78	1.87	0.95	0
24	2.91	2.68	1.08	0
25	1.74	1.64	1.06	2
26	2.20	1.52	1.45	0
27	5.65	4.00	1.41	0
28	2.95	2.72	1.08	2
29	1.99	1.91	1.04	2
30	2.14	2.01	1.06	1
31	1.82	1.83	0.99	0
32	1.84	1.90	0.97	0
33	2.92	1.73	1.69	0
34	1.87	1.90	0.98	2
35	2.41	1.54	1.56	0
36	1.87	1.90	0.98	2
37	1.90	1.80	1.06	0
38	1.88	1.76	1.07	1
39	2.56	2.22	1.15	1

Sup.Tab. 1: Absolute mdm2/CEN12 FISH hybridization signals / cell nucleus (column 2 and 3) and signal ratios (column 4) seen in tumor tissues derived from abemaciclib treated patients Additionally, HER4 IHC staining scores (column 5) are listed. n.a.= not analyzable.
